# Modulation of the Gut Microbiota during High-Dose Glycerol Monolaurate-Mediated Amelioration of Obesity in Mice Fed a High-Fat Diet

**DOI:** 10.1128/mBio.00190-20

**Published:** 2020-04-07

**Authors:** Minjie Zhao, Zengliang Jiang, Haiying Cai, Yang Li, Qiufen Mo, Lingli Deng, Hao Zhong, Tao Liu, Hui Zhang, Jing X. Kang, Fengqin Feng

**Affiliations:** a College of Biosystems Engineering and Food Science, Zhejiang University, Hangzhou, China; b National Engineering Laboratory of Intelligent Food Technology and Equipment, Zhejiang University, Hangzhou, China; c Key Laboratory for Agro-Products Postharvest Handling of Ministry of Agriculture, Zhejiang University, Hangzhou, China; d Key Laboratory for Agro-Products Nutritional Evaluation of Ministry of Agriculture, Zhejiang University, Hangzhou, China; e Zhejiang Key Laboratory for Agro-Food Processing, Zhejiang University, Hangzhou, China; f Institute of Basic Medical Sciences, School of Life Sciences, Westlake University, Hangzhou, China; g Zhejiang Key Lab for Chem & Bio Processing Technology of Farm Product, Zhejiang University of Science and Technology, Hangzhou, China; h College of Food Science and Engineering, Qingdao Agricultural University, Qingdao, China; i Laboratory of Lipid Medicine and Technology, Department of Medicine, Massachusetts General Hospital, Boston, Massachusetts, USA; j Harvard Medical School, Boston, Massachusetts, USA; Brigham and Women's Hospital, Harvard Medical School; Rutgers, The State University of New Jersey

**Keywords:** glycerol monolaurate, obesity, gut microbiota, serum metabolome, hepatic transcriptome

## Abstract

Obesity and associated metabolic disorders are worldwide public health issues. The gut microbiota plays a key role in the pathophysiology of diet-induced obesity. Glycerol monolaurate (GML) is a widely consumed food emulsifier with antibacterial properties. Here, we explore the anti-obesity effect of GML (1,600 mg/kg of body weight) in high-fat diet (HFD)-fed mice. HFD-fed mice were treated with 1,600 mg/kg GML. Integrated microbiome, metabolome, and transcriptome analyses were used to systematically investigate the metabolic effects of GML, and antibiotic treatment was used to assess the effects of GML on the gut microbiota. Our data indicated that GML significantly reduced body weight and visceral fat deposition, improved hyperlipidemia and hepatic lipid metabolism, and ameliorated glucose homeostasis and inflammation in HFD-fed mice. Importantly, GML modulated HFD-induced gut microbiota dysbiosis and selectively increased the abundance of Bifidobacterium pseudolongum. Antibiotic treatment abolished all the GML-mediated metabolic improvements. A multiomics (microbiome, metabolome, and transcriptome) association study showed that GML significantly modulated glycerophospholipid metabolism, and the abundance of Bifidobacterium pseudolongum strongly correlated with the metabolites and genes that participated in glycerophospholipid metabolism. Our results indicated that GML may be provided for obesity prevention by targeting the gut microbiota and regulating glycerophospholipid metabolism.

## Abstract (precis)

Obesity and associated metabolic disorders are worldwide public health issues. The gut microbiota plays a key role in the pathophysiology of diet-induced obesity. Glycerol monolaurate (GML) is a widely consumed food emulsifier with antibacterial properties. Here, we explore the anti-obesity effect of GML (1,600 mg/kg of body weight) in high-fat diet (HFD)-fed mice. HFD-fed mice were treated with 1,600 mg/kg GML. Integrated microbiome, metabolome, and transcriptome analyses were used to systematically investigate the metabolic effects of GML, and antibiotic treatment was used to assess the effects of GML on the gut microbiota.

## INTRODUCTION

The global prevalence of obesity and related metabolic comorbidities has increased considerably over the past several decades (1). The consumption of a high-fat diet (HFD) is one of the major causes of obesity and associated metabolic diseases (2). Excessive body weight and visceral lipid accumulation are major characteristics of obesity, which further results in chronic, low-grade systemic inflammation and disturbed glucose and lipid metabolism, leading to metabolic syndrome, nonalcoholic fatty liver disease (NAFLD), type 2 diabetes, and cardiovascular diseases (3, 4). Growing evidence has implicated the gut microbiota as a crucial player in the pathogenesis of diet-induced obesity and related metabolic complications (5, 6). Gut microbiota dysbiosis induced by an HFD participates in obesity development through different mechanisms, including energy homeostasis, gut permeability, and inflammatory and immune reactions (7, 8). Importantly, gut bacterium-derived metabolites, such as lipopolysaccharide (LPS), bile acids, short-chain fatty acids, and hydrogen sulfide (H_2_S), are also involved in the metabolic effects of gut microbiota on the host (9–12). The binding of LPS to LPS-binding protein (LBP) and its transfer to the receptor CD14 can initiate the secretion of proinflammatory cytokines and maintain the metabolic inflammation (13). Reconstructing gut microbiotas might be an effective strategy to prevent obesity and related metabolic disorders.

Glycerol monolaurate (GML), a 1-monoglyceride of lauric acid, exists naturally in coconut fat, palmetto oil, and breast milk and is recognized as a generally recognized as safe (GRAS) food emulsifier by the U.S. Food and Drug Administration (FDA). GML has been widely used in various foods, such as meat products and bakery products, and thus, the general public has been extensively exposed to GML (14, 15). With great antibacterial and antiviral properties, GML can effectively prevent simian immunodeficiency virus (SIV) transmission and postoperative infection (16, 17). Additionally, GML alters the lipid dynamics in human T cells and prevents cytokine production and exotoxin stimulation, suggesting that it has immunomodulatory functions (18, 19). Our previous results demonstrated that GML administration at 150 mg/kg of body weight with a low-fat diet (LFD, 10% kcal from fat) caused significant increases in body weight and fat deposition and induced gut microbiota dysbiosis (20).

In another study, we found that a higher dose of GML (1,600 mg/kg) significantly decreased chronic systemic inflammation and modulated gut microbiota in LFD-fed mice (21). In the present study, we examined the effects of 1,600 mg/kg GML supplementation on physiology and gut microbiota in HFD-fed mice. Our results indicated that high-dose GML ameliorated weight gain, hyperlipidemia, hepatic steatosis, glucose homeostasis, and systemic inflammation. These effects disappeared after antibiotic treatment, indicating that the gut microbiota played a key role in GML-induced metabolic improvements. Integrative analysis of the metabolome and transcriptome revealed that GML improved lipid metabolism by regulating glycerophospholipid metabolism, enhancing lipid β-oxidation, and modulating cholesterol metabolism, which were associated with the altered gut microbiota composition. Our results provide novel insights into the role of GML in preventing gut microbiota dysbiosis, diet-induced obesity, and related metabolic disorders.

## RESULTS

### GML supplementation prevents HFD-induced obesity in mice.

**FIG 1:**
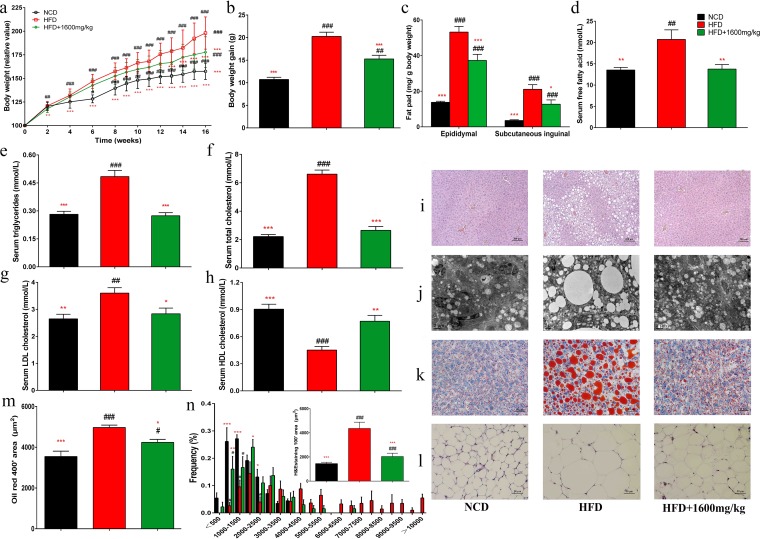
GML reduced body weight, fat accumulation, and serum hyperlipidemia in high-fat diet (HFD)-fed mice. Mice were fed a normal chow diet (NCD), an HFD, or an HFD with 1,600 mg/kg GML (HFD+1600mg/kg) for 16 weeks. (a) Relative body weight curves. (b) Total weight gain. (c) Epididymal and subcutaneous inguinal fat weight. GML administration to HFD-fed mice prevented diet-induced increases in serum free fatty acid (d), triglycerides (e), total cholesterol (f), and low-density lipoprotein cholesterol (LDLC) (g) and decreased serum levels of high-density lipoprotein cholesterol (HDLC) (h). The liver lipid content was assessed using H&E staining (i), Oil Red O staining (j), and transmission electron microscopy (TEM) (k). (l) Epididymal adipocyte size was assessed by H&E staining. (m and n) The liver lipid size in panel k and the adipocyte size in panel l were estimated using the ImageJ software. Numbers of mice tested were 14 to 15 (a to h) and 8 to 10 (i to n). Data are expressed as means ± SEM. Values with asterisks and pound symbols are significantly different based on one-way analysis of variance (ANOVA) with Tukey’s *post hoc* test (*, *P* < 0.05 versus HFD controls; **, *P < *0.01 versus HFD controls; ***, *P < *0.001 versus HFD controls; #, *P < *0.05 versus NCD controls; ##, *P < *0.01 versus NCD controls; ###, *P < *0.001 versus NCD controls).

Administration of GML at 1,600 mg/kg to HFD-fed mice significantly prevented weight gain (*P < *0.001) and fat accumulation in epididymal and subcutaneous inguinal adipose tissues (*P < *0.001 and *P = *0.049, respectively) (Fig. 1a to c). The effects of GML supplementation on hepatic steatosis were shown by hematoxylin and eosin (H&E) staining, Oil Red staining, and transmission electron microscopy (TEM) analysis (Fig. 1i to k), and GML significantly prevented lipid deposition in hepatocytes (Fig. 1m) (*P = *0.049). Additionally, GML significantly reduced the size of epididymal adipocytes (Fig. 1l and n) (*P < *0.001). Moreover, GML protected against HFD-induced hyperlipidemia, significantly decreased the serum concentrations of triglycerides (TG), total cholesterol (TC), low-density lipoprotein cholesterol (LDLC), and free fatty acids (FFA) (*P < *0.001, *P < *0.001, *P = *0.030, and *P = *0.007, respectively), and elevated the level of high-density lipoprotein cholesterol (HDLC) (*P = *0.002) (Fig. 1d to h). GML treatment did not induce any influence on energy intake (see Fig. S1 in the supplemental material), implying that the effects of GML on body weight, serum lipid metabolism, and other obesity-related parameters were independent of the reduction in food consumption. The lipid profile analysis of feces also indicated that GML treatment had no significant effect on the fecal total-lipid content of HFD-fed mice (Table S1). These results together demonstrate the ability of GML to ameliorate HFD-induced weight gain, visceral fat accumulation, and serum lipid profiles.

10.1128/mBio.00190-20.1 FIG S1 Total energy intake is not significantly altered by GML supplementation (15 mice were tested for each group). Download FIG S1, DOCX file, 0.1 MB. Copyright © 2020 Zhao et al. 2020 Zhao et al. This content is distributed under the terms of the Creative Commons Attribution 4.0 International license.

10.1128/mBio.00190-20.7 TABLE S1 Lipid profile of feces of the experimental groups. HFD+1600mg/kg, mice fed a high-fat diet with 1,600 mg/kg GML. Values with asterisks and pound symbols are significantly different based on a one-way analysis of variance (ANOVA) with Tukey’s *post hoc* test (*, *P* < 0.05 versus HFD controls; **, *P < *0.01 versus HFD controls; ***, *P < *0.001 versus HFD controls; #, *P < *0.05 versus NCD controls; ##, *P < *0.01 versus NCD controls; ###, *P < *0.001 versus NCD controls) (*n* = 8). Download Table S1, DOCX file, 0.02 MB. Copyright © 2020 Zhao et al. 2020 Zhao et al. This content is distributed under the terms of the Creative Commons Attribution 4.0 International license.

### GML supplementation improves insulin resistance and reduces metabolic endotoxemia in HFD-fed mice.

**FIG 2:**
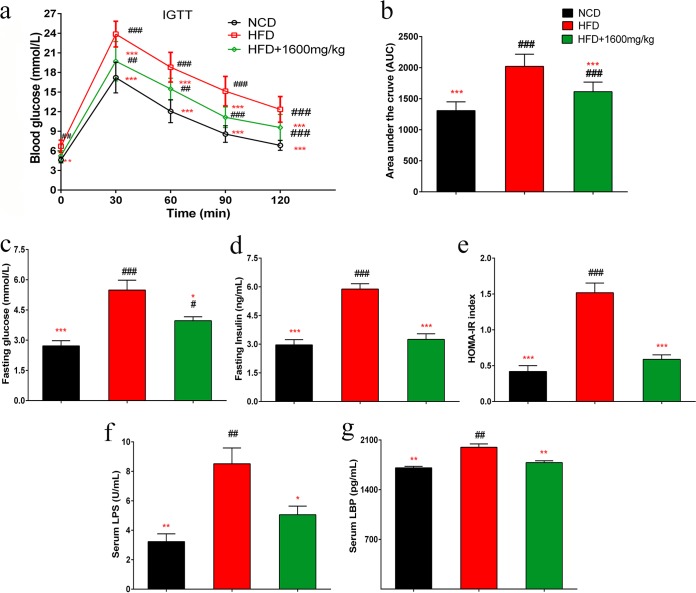
GML supplementation increased insulin sensitivity, improved insulin resistance, and reduced circulating lipopolysaccharide (LPS) and LPS-binding protein (LBP) levels in HFD-fed mice. Mice were fasted overnight (12 h). (a) An intraperitoneal injection glucose tolerance test (IGTT) was performed (2 g/kg body weight). (b) Areas under the curve (AUCs) of the IGTTs. Mice were fasted for 12 h for fasting serum glucose (c) and fasting serum insulin determination (d). (e) HOMA-IR was calculated with the following formula: fasting glucose (millimoles/liter) × fasting insulin (milliunits/liter)/22.5. GML-treated mice showed significantly reduced circulating LPS (f) and LBP (g) levels compared with those of HFD controls. (a to g) Fourteen to 15 mice were tested. Data are expressed as means ± SEM. Values with asterisks and pound symbols are significantly different based on a one-way ANOVA with Tukey’s *post hoc* test (*, *P* < 0.05 versus HFD controls; **, *P < *0.01 versus HFD controls; ***, *P < *0.001 versus HFD controls; #, *P < *0.05 versus NCD controls; ##, *P < *0.01 versus NCD controls; ###, *P < *0.001 versus NCD controls).

An intraperitoneal glucose tolerance test (IGTT) was performed, and the calculated area under the curve (AUC) of the IGTT result was significantly decreased in the GML-treated group compared with that of the HFD control (Fig. 2a and b) (*P < *0.001). Additionally, GML-treated HFD-fed mice showed improved fasting glycemia and fasting insulin (Fig. 2c and d) (*P = *0.012 and *P < *0.001, respectively), resulting in a remarkable reduction in the homeostasis model assessment of insulin resistance (HOMA-IR) index (Fig. 2e) (*P < *0.001). These results indicate that GML supplementation markedly improved glucose homeostasis.

LPS can modulate the signaling pathways of TLR4, which is related to the production of proinflammatory cytokines and insulin resistance (22–24). Sixteen weeks of HFD feeding produced a >2-fold increase in the circulating LPS load, which was significantly decreased by GML supplementation (Fig. 2f) (*P = *0.021). This observation was supported by a lower LBP concentration in GML-treated HFD-fed mice (Fig. 2g) (*P =* 0.003). Taken together, these results suggest that GML treatment can effectively attenuate the endotoxemia load, suggesting its potential in alleviating systemic inflammation.

### GML supplementation ameliorates systemic inflammation in HFD-fed mice.

**FIG 3:**
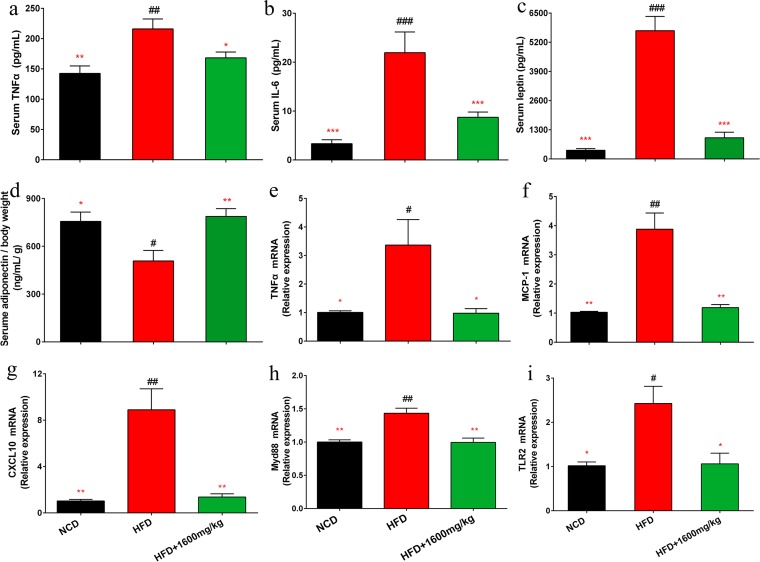
GML supplementation improved systemic inflammation in HFD-fed mice. GML treatment reduced the levels of serum proinflammatory cytokines TNF-α (a) and IL-6 (b) and increased the levels of the serum adipocytokines leptin (c) and adiponectin (d) corrected for body weight in HFD-fed mice. The relative mRNA expression levels of the inflammation-related genes encoding TNF-α (e), MCP-1 (f), CXCL10 (g), Myd88 (h), and TLR2 (i) in hepatic tissue were assessed using qRT-PCR. Fourteen to 15 mice for panels a to d and 8 to 10 mice for panels e to i were tested. Data are expressed as means ± SEM. Values with asterisks and pound symbols are significantly different based on a one-way ANOVA with Tukey’s *post hoc* test (*, *P* < 0.05 versus HFD controls; **, *P < *0.01 versus HFD controls; ***, *P < *0.001 versus HFD controls; #, *P < *0.05 versus NCD controls; ##, *P < *0.01 versus NCD controls; ###, *P < *0.001 versus NCD controls).

GML treatment in HFD-fed mice significantly reduced the circulating concentrations of the proinflammatory cytokines tumor necrosis factor alpha (TNF-α) and interleukin 6 (IL-6) (*P = *0.039 and *P < *0.001, respectively) (Fig. 3a and b). GML effectively reduced the serum leptin level and ameliorated HFD-induced leptin resistance (Fig. 3c) (*P < *0.001). Adiponectin concentrations of GML-treated HFD-fed mice were significantly elevated, implying an improved anti-inflammatory action (Fig. 3d) (*P = *0.006). The expression of hepatic TNF-α was upregulated by the HFD, which was reversed by GML supplementation (Fig. 3e) (*P = *0.020). Additionally, GML significantly decreased the expression levels of MCP-1 and CXCL10, which are indicators of macrophage infiltration (Fig. 3f and g) (*P < *0.001 and *P < *0.001, respectively). MyD88, a key downstream signaling molecule of Toll-like receptors (TLRs), is required for TLR2-mediated responses to inflammation (25). MyD88 activation plays an important role in the stimulation of inflammation by LPS (26). GML reversed the remarkable upregulation of hepatic MyD88 and TLR2 expression induced by the HFD (Fig. 3h and i) (*P < *0.001 and *P = *0.029, respectively). These results imply that the effects of GML treatment are anti-inflammatory.

### GML alleviates HFD-induced hepatic steatosis.

**FIG 4:**
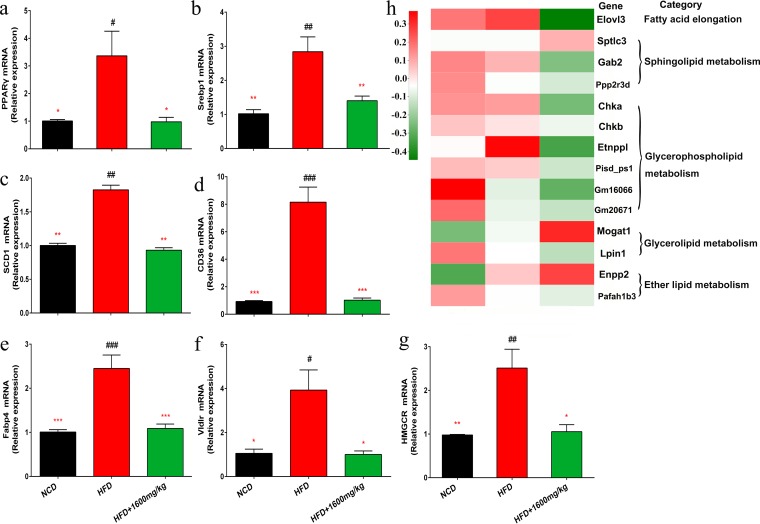
GML supplementation regulated hepatic lipid metabolism in HFD-fed mice. Using qRT-PCR, we assessed hepatic expression levels of genes related to lipid metabolism and participating in the PPARγ or Srebp1 pathway, including PPARγ (a), Srebp1 (b), SCD1 (c), CD36 (d), Fabp4 (e), Vldlr (f), and HMGCR (g). The hepatic transcriptome identified the DEGs between the GML-treated group and the HFD controls. (h) A heatmap demonstrated that these DEGs were enriched in lipid metabolism-related pathways. Eight to 10 mice were tested for panels a to g, and 4 mice were tested for panel h. Data are expressed as means ± SEM. Values with asterisks and pound symbols are significantly different based on a one-way ANOVA with Tukey’s *post hoc* test (*, *P* < 0.05 versus HFD controls; **, *P < *0.01 versus HFD controls; ***, *P < *0.001 versus HFD controls; #, *P < *0.05 versus NCD controls; ##, *P < *0.01 versus NCD controls; ###, *P < *0.001 versus NCD controls).

GML supplementation significantly prevented hepatic upregulation of peroxisome proliferator-activated receptor γ (PPARγ) in HFD-fed mice (*P = *0.020) and modulated the expression of related genes in the PPARγ signaling pathway (Fig. 4). Notably, sterol regulatory element binding transcription factor 1 (Srebp1) was significantly downregulated after GML treatment of HFD-fed mice (Fig. 4b) (*P = *0.006). PPARγ and Srebp1 modulate the expression of the lipogenesis gene for stearoyl-coenzyme A (CoA) desaturase 1 (SCD1) and fatty acid transport-related gene CD36 (27, 28), and our results indicated that GML significantly reversed the elevated expression of SCD1 and CD36 in mice fed a HFD (Fig. 4c and d) (*P = *0.002 and *P < *0.001, respectively). Upregulated hepatic PPARγ is related to an increased expression of fatty acid binding protein 4 (Fabp4) and the very-low-density lipoprotein receptor (Vldlr) (27, 29), which was effectively modified by GML treatment, consistent with lower levels of serum FFA and TG (Fig. 4e and f) (*P < *0.001 and *P = *0.015, respectively). Additionally, GML efficiently downregulated the expression level of the rate-limiting enzyme hydroxymethylglutaryl-CoA reductase (HMGCR) in cholesterol synthesis, supported by a lower serum cholesterol level in the GML-treated group (Fig. 4g) (*P = *0.022). Markedly, the results of hepatic transcriptome analysis demonstrated that the expression levels of those genes involved in fatty acid elongation, sphingolipid metabolism, glycerophospholipid metabolism, glycerolipid metabolism, and ether lipid metabolism were significantly different between the HFD control and GML-treated groups (Fig. 4h). These data indicated that GML affected hepatic lipid metabolism in HFD-fed mice by inhibiting the PPARγ and Srebp1 signaling pathways.

### GML treatment modifies the gut microbiota composition in HFD-induced mice.

**FIG 5:**
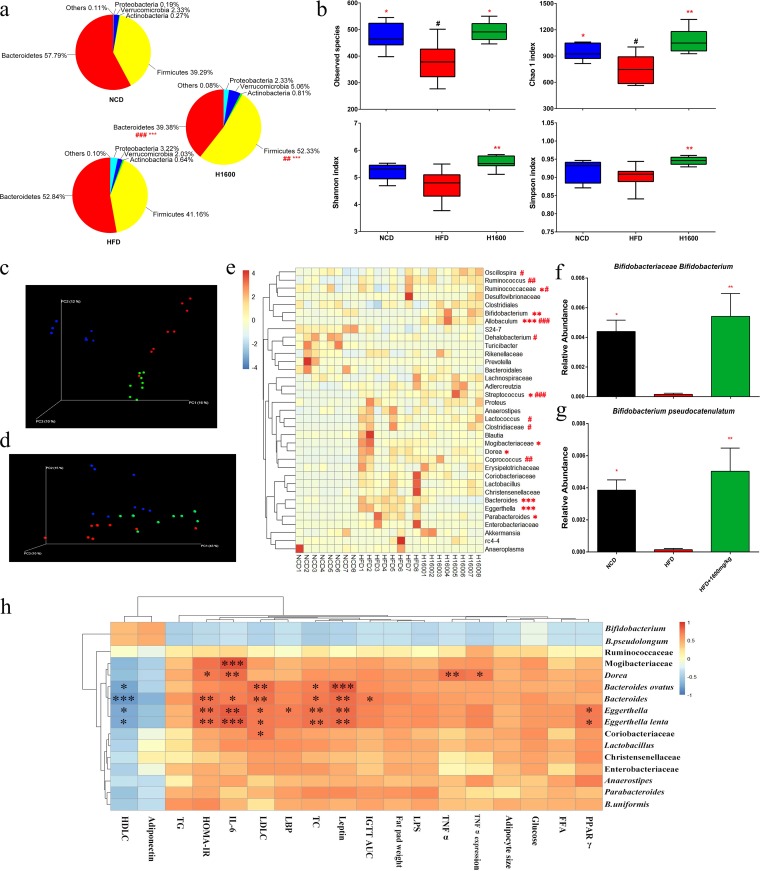
GML supplementation altered the diversity and composition of the gut microbiota and its correlation with metabolic improvements in HFD-fed mice. (a) Relative abundances of gut microbes at the phylum level. (b) α-Diversities of observed species, Chao 1 index, Shannon index, and Simpson index. (c) Unweighted UniFrac PCoA plot based on operational taxonomic unit (OTU) abundance. (d) Weighted UniFrac PCoA plot based on OTU abundance. (e) Relative abundances of gut microbes at the family and genus levels (those with > 0.1% are represented) and species in the GML-treated group at week 16, including *Bifidobacteriaceae*, *Bifidobacterium* (f), and Bifidobacterium pseudocatenulatum (g), that were significantly changed from those in HFD controls. (h) Correlations between GML-induced significantly changed gut microbes at the family, genus, and species levels and host obesity-related metabolic parameters. Eight mice were tested for panels a to h. Data are expressed as means ± SEM. Values with asterisks and pound symbols are significantly different based on the nonparametric factorial Kruskal-Wallis test, as well as by the unpaired Wilcoxon comparison test (*, *P* < 0.05 versus HFD controls; **, *P < *0.01 versus HFD controls; ***, *P < *0.001 versus HFD controls; #, *P < *0.05 versus NCD controls; ##, *P < *0.01 versus NCD controls; ###, *P < *0.001 versus NCD controls; in panel h, significant correlations are marked by * [*P* < 0.05], ** [*P < *0.01], and *** [*P < *0.001]).

Analysis at the phylum level indicated that the gut microbiota was dominated by five major phyla: *Bacteroidetes*, *Firmicutes*, *Verrucomicrobia*, *Proteobacteria*, and *Actinobacteria*. Compared with mice in the normal chow diet (NCD) group, HFD-fed mice showed an increase in numbers of *Firmicutes* and a reduction in numbers of *Bacteroidetes* (Fig. 5a). Interestingly, GML significantly increased *Firmicutes* and decreased *Bacteroidetes* numbers compared to numbers in both the HFD and NCD groups. Additionally, GML supplementation showed a trend toward decreased numbers of *Proteobacteria* and increased numbers of *Verrucomicrobia* (Fig. 5a). For α-diversity analysis, the number of observed species, the Chao 1 index, and the Shannon and Simpson indexes were significantly increased by GML treatment (Fig. 5b) (*P = *0.013, *P = *0.009, *P = *0.002, and *P = *0.004, respectively). The β-diversity was also profoundly altered in the GML-supplemented group. Clear separations between the communities were observed, especially in the unweighted UniFrac indexes, which indicated overall structural changes of gut microbiota after GML treatment (Fig. 5c and d). Detailed analyses showed that numbers of *Mogibacteriaceae* were significantly decreased in GML-supplemented HFD-fed mice at the family level (Fig. 5e) (*P = *0.045). At the genus level, numbers of *Dorea*, *Bacteroides*, *Eggerthella*, and *Parabacteroides* organisms were significantly lower in HFD-fed mice treated with GML than in untreated mice (Fig. 5e) (*P = *0.041, *P < *0.001, *P < *0.001, *P = *0.022, respectively). Moreover, GML markedly increased numbers of *Bifidobacterium*, *Allobaculum*, and *Streptococcus* organisms compared to numbers in the HFD-fed control (Fig. 5e) (*P = *0.004, *P < *0.001, and *P = *0.033, respectively). Clustering analysis using system-theoretic accident model and processes (STAMP) revealed marked changes in gut microbiota compositions at the species level (Fig. S2). Notably, in the GML-treated group, the abundance of Bifidobacterium pseudolongum was significantly increased (Fig. 5g) (*P = *0.003). Collectively, these results indicate that GML modulates the gut microbial composition in HFD-fed mice, resulting in a gut microbiota composition similar to that of NCD-fed mice.

10.1128/mBio.00190-20.2 FIG S2 STAMP analysis of gut microbiota composition at the species level among mice fed an NCD, HFD, and HFD plus 1,600 ppm GML (H1600). (a) PCA plot comparing genus-level taxonomic profiles (*n* = 10 for each group). Plots showing significant differences in the abundances of species between NCD and HFD groups (b), HFD and H1600 groups (c), and NCD and H1600 groups (d). The dot plots on the right side display the differences in mean proportions between the two groups compared, with an associated *P* value; the bar graphs on the left side demonstrate the mean proportion of a genus in each group. Download FIG S2, DOCX file, 0.3 MB. Copyright © 2020 Zhao et al. 2020 Zhao et al. This content is distributed under the terms of the Creative Commons Attribution 4.0 International license.

Furthermore, the correlation analysis of gut bacterial genera/species and the markers of obesity and related metabolic complications indicated that the abundances of the *Dorea*, *Bacteroides*, and *Eggerthella* genera, *B. ovatus*, and *E. lenta* were significantly positively associated with some parameters related to metabolic disorders (Fig. 5h). These bacterial taxa were all less abundant in GML-treated HFD-fed mice. These results suggest that the improvements in HFD-induced obesity by GML supplementation were associated with significant changes in the gut microbiota composition.

### GML-induced improvements in lipid metabolism partially depend on the gut microbiota.

**FIG 6:**
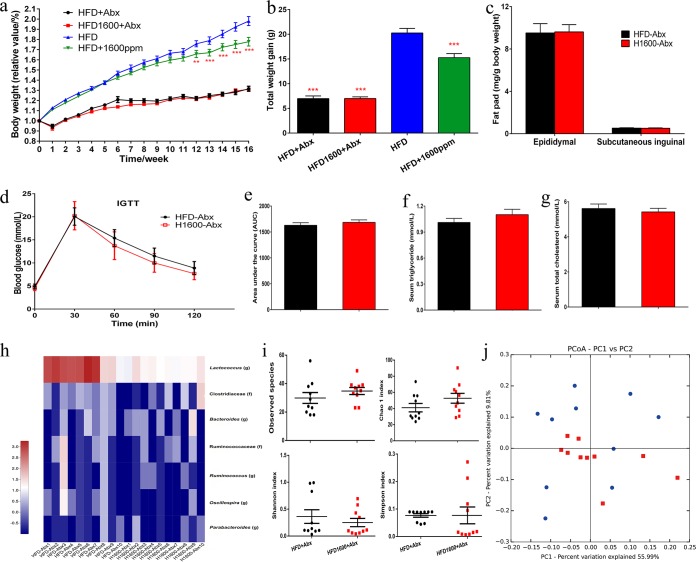
Continuous antibiotic treatment abrogated the metabolic improvement induced by GML supplementation in HFD-fed mice. Mice were fed an HFD supplemented with antibiotic treatment and given (H1600-Abx) or not given (HFD-Abx) 1,600 mg/kg GML for 16 weeks. The GML-treated group exhibited no significant difference in body weight curves (a), total weight gain (b), or fat pad weight (c) from those of HFD controls under antibiotic treatment. Insulin sensitivity also demonstrated no significant change between these two groups. (d) Mice were fasted overnight (12 h) for an IGTT (2 g/kg body weight). (e) The AUCs of the IGTTs were evaluated. Serum hyperlipidemia, including the levels of serum triglyceride (f) and serum total cholesterol (g), was not significantly altered. Additionally, continuous antibiotic treatment also abolished the gut microbiota alterations induced by GML supplementation. (h) Heatmap demonstrating the abundances of detected bacterial taxa which were significantly changed by GML treatment in HFD-fed mice after antibiotic treatment. (i) α-Diversities for observed species, Chao 1 indexes, Shannon indexes, and Simpson indexes. (j) Unweighted UniFrac PCoA plot based on OTU abundance. Fourteen to 15 mice (a to g) and 10 mice (h to j) were tested. Data are expressed as means ± SEM. Values with asterisks are significantly different based on a one-way analysis of variance with Tukey’s *post hoc* test (*, *P* < 0.05 versus HFD controls; **, *P < *0.01 versus HFD controls; ***, *P < *0.001 versus HFD controls).

After 16 weeks of continuous antibiotic treatment, body weight, total body weight gain, and the relative weights of fat pads displayed no differences between the groups fed an HFD with antibiotics (HFD-Abx) and an HFD with GML and Abx (HFD-GML-Abx) (Fig. 6a to c). Furthermore, the AUC of the IGTT and serum HOMA-IR did not change with GML supplementation (Fig. 6d and e). Concomitantly, GML supplementation exerted no significant effects on the serum lipid profile (Fig. 6f and g; Fig. S3) or on the serum levels of TNF-α, IL-6, LPS, and LBP after antibiotic treatment (Fig. S3). 16S rRNA analysis revealed that the shifts in gut microbiota composition observed in GML-treated mice were diminished (Fig. 6h to j; Fig. S4). The genus and species that were significantly affected by GML were undetectable or showed no difference in abundance between GML-treated and untreated HFD-fed mice after antibiotic treatment (Fig. 6h). There was no significant difference in α-diversity and β-diversity between the HFD-Abx and HFD-GML-Abx groups (Fig. 6i and j). Based on the above results, the improvements in lipid metabolism, systematical inflammation, and glucose homeostasis induced by GML supplementation partially depended on its modulation of the gut microbiota.

10.1128/mBio.00190-20.3 FIG S3 Continuous antibiotic treatment abrogated the metabolic improvement induced by GML supplementation in HFD-fed mice. Mice were fed a HFD supplemented with (H1600-Abx) or without 1600 mg/kg GML (HFD-Abx) under antibiotic treatment for 16 weeks. (a) Continuous antibiotics treatment significantly reduced the abundance of gut microbes, supported by the DNA concentrations in fecal samples (*n* = 10 for each group). The GML-treated group exhibited no significant difference in (b) serum HDLC, (c) serum LDLC, (d) serum LPS, (e) serum LBP, (f) serum IL-6, and (g) serum TNF-α, *n* = 14-15 (b-g). Data are expressed as the mean ± SEM. Values with asterisks are significantly different based on one-way analysis of variance with Tukey’s post hoc test (*, *P* < 0.05 versus HFD controls; **, *P* < 0.01 versus HFD controls; ***, *P* < 0.001 versus HFD controls). Download FIG S3, DOCX file, 0.2 MB. Copyright © 2020 Zhao et al. 2020 Zhao et al. This content is distributed under the terms of the Creative Commons Attribution 4.0 International license.

### GML supplementation reverses the effects of an HFD on serum metabolomics.

**FIG 7:**
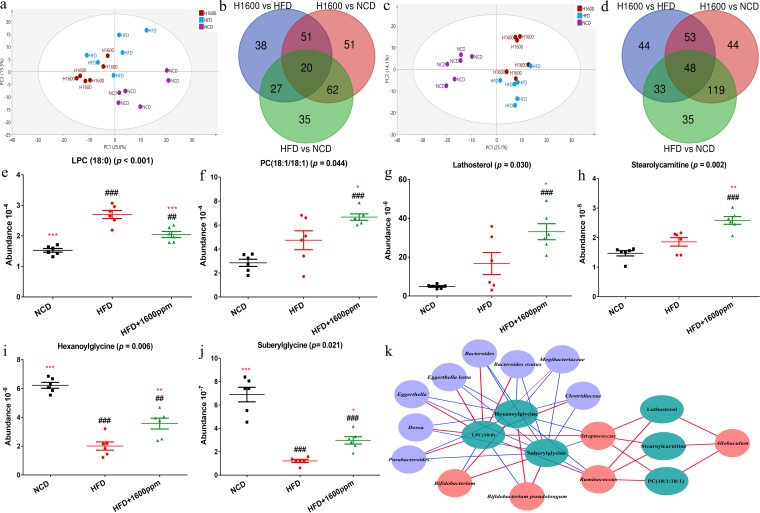
Serum metabonomics analysis after GML supplementation. (a) PCA plot based on the metabolites detected in positive electrospray ionization (ESI+) mode. (b) Venn diagram of characterized metabolites in two groups’ comparisons in ESI+ mode. (c) PCA plot based on the metabolites detected in negative electrospray ionization (ESI−) mode. (d) Venn diagram of characterized metabolites in two groups’ comparisons in the ESI− mode. (e to j) Six serum lipid metabolites, including LPC(18:0) (e), PC(18:1/18:1) (f), lathosterol (g), stearoylcarnitine (h), hexanoylglycine (i), and suberylglycine (j), were significantly altered by GML treatment. (k) Correlation network between GML-induced significant changes in gut microbiotas at the genus and species levels and 6 significantly changed serum lipid metabolites (those for which *P *was <0.05 are represented, and detailed Spearman correlation indexes are listed in Table S3 in the supplemental material). (a to i) Six mice were tested. Data are expressed as means ± SEM. Values with asterisks and pound symbols are significantly different based on a one-way ANOVA with Tukey’s *post hoc* test (*, *P* < 0.05 versus HFD controls; **, *P < *0.01 versus HFD controls; ***, *P < *0.001 versus HFD controls; #, *P < *0.05 versus NCD controls; ##, *P < *0.01 versus NCD controls; ###, *P < *0.001 versus NCD controls).

To reveal the effects of GML supplementation on the metabolic phenotypes of HFD-fed mice, we performed serum metabolomics profiling by ultrahigh-performance liquid chromatography–quadrupole time of flight mass spectrometry (UHPLC-Q-TOF-MS). A principal-component analysis (PCA) for each of the acquired modes (electrospray ionization negative/electrospray ionization positive [ESI–/ESI+]) demonstrated that HFD feeding induced a serum metabolomics profile distinct from that obtained with NCD feeding, whereas the GML-treated group exhibited a strong separation from HFD-fed mice in each mode (Fig. 7a and c). Orthogonal projection to latent structure discriminant analysis (OPLS-DA) models were built to distinguish each experimental group in pairwise comparisons, and permutation analyses were demonstrated to validate the models (Fig. S5 and S6). The characterized metabolites that displayed significant differences between the two groups (HFD versus HFD plus 1,600 mg/kg GML, NCD versus HFD plus 1,600 mg/kg GML, HFD versus NCD) are highlighted in red in Fig. S5 and S6. Among the characterized metabolites from two groups’ comparisons, 48 in the ESI– model and 20 in the ESI+ model were common differential metabolites in all three groups (Fig. 7b and d). Based on the results of one-way analysis of variance (ANOVA), the lipid metabolites significantly affected by GML are shown in Fig. 7e to j. Among these significantly increased metabolites (Fig. 7f to h), 1,2-dioleoyl-phosphatidylcholine 18:1/18:1 [PC(18:1/18:1)] (*P = *0.044) is involved in glycerophospholipid metabolism and linoleic acid metabolism, lathosterol (*P = *0.030) participates in endogenous cholesterol, and stearoylcarnitine (*P = *0.002) is related to mitochondrial beta-oxidation of long-chain saturated fatty acids. Among the most decreased metabolites (Fig. 7e and i to j), 1-stearoyl-sn-glycerol-3-phosphocholine {lysophosphatidylcholine 18:0 [LPC(18:0)]} (*P < *0.001) also participates in glycerophospholipid metabolism, and hexanoylglycine (*P = *0.002) and suberylglycine (*P = *0.002) are acylglycines and lipid β-oxidation intermediates.

10.1128/mBio.00190-20.4 FIG S4 Continuous antibiotic treatment abolished the gut microbiota alterations induced by GML supplementation. (a) Continuous antibiotic treatment significantly reduced the abundance of gut microbes, supported by the DNA concentrations in fecal samples (*n* = 10 for each group). (b) Heatmap demonstrating the abundances of detected bacterial taxa which were significantly changed by GML treatment of HFD-fed mice after antibiotic treatment. Data are expressed as means ± SEM. Values with asterisks are significantly different based on the results of a one-way analysis of variance with Tukey’s *post hoc* test (*, *P* < 0.05 versus HFD controls; **, *P < *0.01 versus HFD controls; ***, *P < *0.001 versus HFD controls). Download FIG S4, DOCX file, 0.3 MB. Copyright © 2020 Zhao et al. 2020 Zhao et al. This content is distributed under the terms of the Creative Commons Attribution 4.0 International license.

10.1128/mBio.00190-20.5 FIG S5 Preliminary identifications of potential biomarkers of serum metabolomics in ESI+ mode. Orthogonal projections to a latent structure discriminant analysis (OPLS-DA) model built to discriminate between NCD and HFD groups in ESI+ mode (a to c), between HFD and H1600 groups in ESI+ mode (d to f), and between NCD and H1600 groups in ESI+ mode (g to i). (a, d, g) Score plots; (b, e, h) permutation tests for the goodness-of-fit (*R*^2^) and goodness-of-prediction (*Q*^2^) parameters obtained from a 7-fold-cross-validated OPLS regression model; (c, f, i) variable importance in projection (VIP) loading plots (the red spots represent significantly different metabolites, with a VIP of >1.0 and a *P* [corrected] of >0.5). Download FIG S5, DOCX file, 0.3 MB. Copyright © 2020 Zhao et al. 2020 Zhao et al. This content is distributed under the terms of the Creative Commons Attribution 4.0 International license.

10.1128/mBio.00190-20.6 FIG S6 Preliminary identification of potential biomarkers of serum metabolomics in ESI– mode. OPLS-DA model built to discriminate between NCD and HFD groups in ESI– mode (a to c), between HFD and H1600 groups in ESI– mode (d to f), and between NCD and H1600 groups in ESI– mode (g to i). (a, d, g) Score plots; (b, e, h) permutation tests for the goodness-of-fit (*R*^2^) and goodness-of-prediction (*Q*^2^) parameters obtained from a 7-fold-cross-validated OPLS regression model; (c, f, i) VIP loading plots (the red spots represent significantly different metabolites, with a VIP of >1.0 and a *P* [corrected] of >0.5). Download FIG S6, DOCX file, 0.3 MB. Copyright © 2020 Zhao et al. 2020 Zhao et al. This content is distributed under the terms of the Creative Commons Attribution 4.0 International license.

A network was constructed by the significantly changed serum metabolites and gut microbiota to explain their correlation (Fig. 7k). All microbes that were reduced by GML supplementation in HFD-fed mice showed significantly positive associations with the serum LPC(18:0) level and negative associations with the serum hexanoylglycine and suberylglycine levels. Conversely, Bifidobacterium pseudolongum, which was enriched in the GML-treated group, was negatively correlated with LPC(18:0) and positively correlated with hexanoylglycine and suberylglycine. Additionally, other microbes that responded positively to GML treatment (the *Allobaculum*, *Streptococcus*, and *Ruminococcus* genera) were significantly correlated with high concentrations of serum lathosterol, stearoylcarnitine, and PC(18:1/18:1). Thus, these findings support the modulation of lipid metabolism by GML supplementation in HFD-fed mice, especially with regard to glycerophospholipid and cholesterol metabolism and mitochondrial lipid β-oxidation, suggesting that the regulation of characteristic metabolites is associated with profound changes in gut microbiota composition.

### Correlations among GML-induced gut microbiota, characteristic serum metabolites, and hepatic gene coexpression modules.

**FIG 8:**
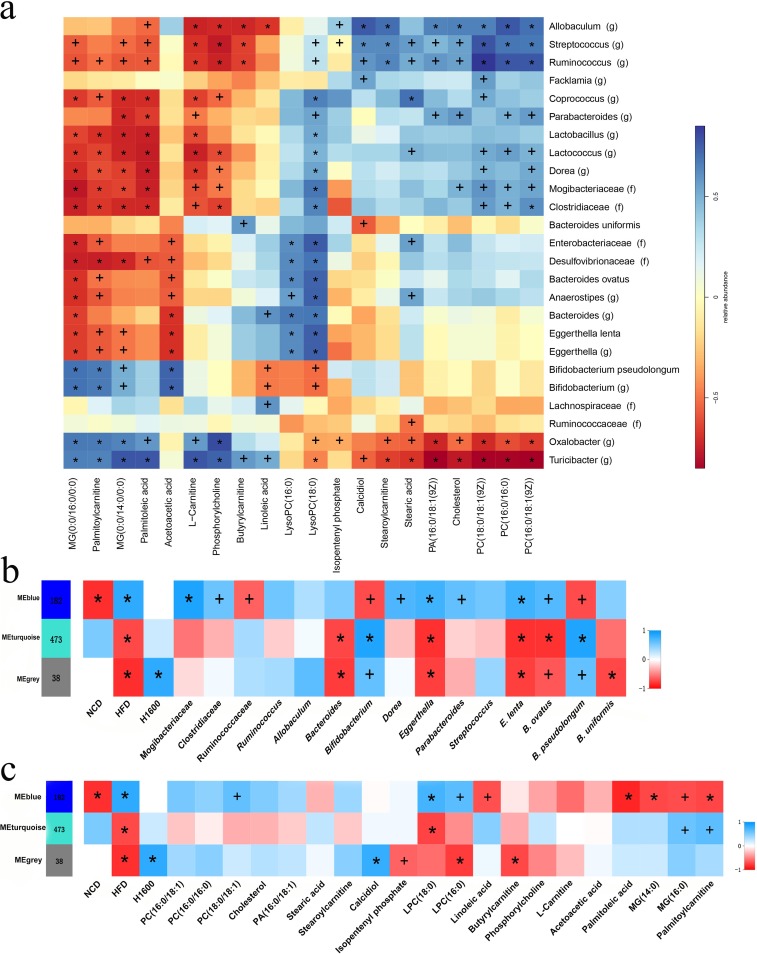
Correlation analyses of serum metabonomics, gut microbiomes, and hepatic transcriptomes. (a) Spearman correlation matrix of the gut microbiota at the genus and species levels and serum metabolites participating in lipid metabolism (the significantly changed bacterial taxa and serum metabolites that were identified by a nonparametric factorial Kruskal-Wallis test and unpaired Wilcoxon comparison test are represented). A parenthetical “g” or “f” indicates genus or family, respectively. (b) A Spearman correlation matrix of a WGCNA identified clusters in hepatic DEGs between the GML-treated group and the HFD controls and gut microbiota at the genus and species levels (only the significantly changed bacterial taxa that were identified by a nonparametric factorial Kruskal-Wallis test and unpaired Wilcoxon comparison test are represented). ME, methylene. (c) A Spearman correlation matrix of WGCNA identified clusters in hepatic DEGs between the GML-treated group and HFD controls and serum metabolites participating in lipid metabolism (only the significantly changed metabolites that were identified by a nonparametric factorial Kruskal-Wallis test and unpaired Wilcoxon comparison test are represented). Six (a) and four (b, c) mice were tested. Significant correlations are marked by + (*P < *0.05) and * (*P < *0.01).

A metabolome-wide association study resulted in 20 serum metabolite signals that were correlated with significantly changed gut microbiota taxa. These related metabolites participate mainly in the following pathways: glycerophospholipid metabolism, cholesterol metabolism, and mitochondrial β-oxidation of long-chain saturated fatty acids (Fig. 8a). Spearman correlation analysis revealed that *Bifidobacterium* and *B. pseudolongum*, showed significantly negative associations with LPC(18:0) (*P = *0.020 and *P = *0.011, respectively), and other metabolites participating in glycerophospholipid metabolism [LPC(16:0), PC(16:0/18:1), PC(16:0/16:0), phosphatidic acid 16:0/18:1 {PA(16:0/18:1)}, PC(18:0/18:1)]. Inversely, these metabolites were positively correlated with the *Dorea* and *Parabacteroides* genera and the *Mogibacteriaceae* and *Clostridiaceae* families. Notably, the abundances of these bacterial taxa were remarkably decreased by GML supplementation compared with their abundances in the HFD control. Linoleic acid, an *n*-6 polyunsaturated fatty acid, was present at a relatively high concentration in the sera of HFD-fed rats (30) and showed negative correlations with the abundances of the *Allobaculum* (*P = *0.003) and *Bifidobacterium* (*P = *0.047) genera and *B. pseudolongum* (*P = *0.036). The increase in serum acetoacetic acid was associated with higher abundances of *Bifidobacterium* (*P < *0.001) and *B. pseudolongum* (*P < *0.001) and lower abundances of the *Bacteroides* (*P = *0.003) and *Eggerthella* (*P = *0.002) genera and *E. lenta* (*P = *0.002). Additionally, palmitoylcarnitine was positively correlated with *Bifidobacterium* (*P = *0.003) and *B. pseudolongum* (*P = *0.002) but negatively associated with the *Dorea* (*P = *0.009) genus and the *Mogibacteriaceae* (*P = *0.004) and *Clostridiaceae* (*P = *0.003) families.

A total of 693 differentially expressed genes (DEGs) were detected when we compared the HFD group and the GML-treated HFD-fed group, and these DEGs were applied to integrated analyses of the hepatic transcriptome with serum metabolomics and the gut microbiota. In total, 3 modules were identified through weighted gene coexpression network analysis (WGCNA) (Fig. 8b and c). These genes in the blue module showed significantly positive correlations with HFD feeding (*P = *0.001) and higher expression levels in HFD-fed mice than in NCD-fed mice. Conversely, the gray and turquoise modules were enriched for genes that were expressed at lower levels in the HFD group. The blue module displayed positive correlation with metabolites in the glycerophospholipid metabolism pathway, especially for LPC(18:0) (*P = *0.007), which showed a lower concentration after GML treatment and whose concentration was negatively correlated with the abundances of *Bifidobacterium* and *B. pseudolongum* organisms (Fig. 8b). The serum metabolites monoacylglyceride 14:0 [MG(14:0)], MG(16:0), and palmitoylcarnitine, which were positively correlated with *Bifidobacterium* and *B. pseudolongum*, showed a significant negative association with the blue module (Fig. 8b) (*P = *0.011, *P = *0.008, and *P = *0.011, respectively). Furthermore, the gut microbial taxa enriched in the GML-treated HFD-fed mice (*Ruminococcaceae*, *Bifidobacterium*, and *B. pseudolongum*) showed a significantly negative correlation with the blue module (Fig. 8c) (*P = *0.031, *P = *0.011, and *P = *0.010, respectively). Conversely, the correlations of the blue module and these bacterial taxa, which were decreased in the GML-treated group compared with levels in the HFD control group (*Mogibacteriaceae*, *Clostridiaceae*, *Coprococcus*, *Dorea*, *Eggerthella*, *Parabacteroides*, *E. lenta*, and *B. ovatus*), were significantly positive (Fig. 8c).

These observations indicated that the changed gut microbiota composition induced by GML was linked to modulated concentrations of characteristic serum metabolites and the regulated expression of hepatic genes. Interestingly, *B. pseudolongum* and serum LPC(18:0) played key roles in GML-induced changes in the gut microbiota, serum metabolites, and hepatic gene expression.

## DISCUSSION

Recently, growing evidence has implicated the crucial role of the gut microbiota in the pathogenesis of diet-induced obesity and related metabolic disorders (5, 6). Chassaing et al. demonstrated that dietary emulsifiers directly affect the gut microbiota and promote low-grade inflammation and metabolic syndrome (31, 32). Our previous research studies have reported that low-dose GML consumption promoted systemic low-grade inflammation in LFD-fed mice (20). However, the effects of GML on the gut microbiota composition, diet-induced obesity, and related metabolic disorders in HFD-fed mice have not been fully investigated. In this study, our results demonstrated that high-dose GML significantly prevented diet-induced obesity, ameliorated lipid metabolism, alleviated systemic inflammation, and improved glucose homeostasis and that these metabolic improvements were associated with the modulation of the gut microbiota by modulating gut microbiota composition. Notably, continuous antibiotic treatment indicated that the metabolic improvements of GML treatment in HFD-fed mice partly depended on modulation of the composition and metabolic function of the gut microbiota.

The liver is a major site for the central control of lipid metabolism (33). PPARγ is a nuclear transcription factor that governs hepatic metabolic pathways and plays a crucial role in TG homeostasis (34). GML treatment significantly decreased the expression of hepatic PPARγ, Srebp1c, and their downstream genes, which are involved in fatty acid and cholesterol synthesis. Such improvements were associated with the attenuation of HFD-induced body weight gain, serum hyperlipidemia, and hepatic steatosis after GML supplementation. The serum metabolomics analysis also supported the amelioration of lipid metabolism by GML. High concentrations of serum LPCs were observed in obese humans and mice, and LPCs have been regarded as major components of atherogenic lipids and positively correlate with inflammation (35, 36). The results of Kim et al. indicated that LPC(18:0) might be a major serum metabolite that contributes to the discrimination between normal mice and HFD-induced obese mice (37). Consequently, the decreased level of serum LPC(18:0) might play an important role in the anti-obesity effect of GML. The significantly changed concentrations of LPC(18:0) and PC(18:1/18:1) implied the potential of GML to regulate glycerophospholipid metabolism. Hexanoylglycine and suberylglycine are obtained from the conjugation of hexanoyl-CoA or suberyl-CoA with glycine (38). The accumulation of hexanoylglycine and suberylglycine by GML treatment indicates an upregulation of fatty acid breakdown in the early stage of β-oxidation in mitochondria and might change energy homeostasis (38, 39). Stearoylcarnitine, a lipid metabolism intermediate, is a long-chain acyl fatty acid derivative ester of carnitine that participates in transportation and mitochondrial β-oxidation of long-chain saturated fatty acids, which regulates fatty acid metabolism and liver function (40). Taken together, GML treatment increased lipid metabolism intermediates (hexanoylglycine, suberylglycine, and stearoylcarnitine), indicating the regulation of lipid and energy metabolism in HFD-fed mice through increased β-oxidation. Lathosterol reflects endogenous cholesterol synthesis, which can be used as a marker of increased endogenous cholesterol synthesis (41). GML supplementation significantly increased the serum lathosterol level but also remarkably decreased the serum TC concentration, suggesting that there might be an increase in cholesterol metabolism. The results of hepatic transcriptomics confirmed the changes in serum metabolites induced by GML treatment, and the lipid metabolism-related DEGs were also involved in glycerophospholipid metabolism. The correlation analysis between serum metabolites and differentially expressed hepatic genes suggests that the lipid metabolism modulation induced by GML treatment was closely associated with glycerophospholipid metabolism, especially the participating metabolite, LPC(18:0). Chen et al. reported that glycerophospholipid metabolism is related to hyperlipidemia and involved in atherogenic changes in metabolic pathways (42).

Obesity is associated with chronic systemic inflammation (43). Our results demonstrated that GML supplementation significantly reduced the concentrations of the serum proinflammatory cytokines IL-6 and TNF-α. The downregulated expression of hepatic MCP-1 and CXCL10 indicated decreased hepatic macrophage infiltration under GML treatment. Adiponectin is regarded as a link between obesity and metabolic disorders and can prevent atherogenesis as an anti-inflammatory factor (44). While HFD-induced reduction in adiponectin was significantly improved by GML supplementation, the sensitivity of another adipocytokine, leptin, was also modulated, which induced hepatic expression of TNF-α and insulin resistance (45). These results suggested that adipose tissue might also be the target for the anti-obesity effects of GML, which requires further study. The gut microbiota has also been linked to chronic metabolic inflammation in HFD-fed mice (46). LPS, a component of the cell walls of Gram-negative bacteria, can induce proinflammatory responses in the host by activating TLR2 (43). GML decreased the levels of circulating LPS and LBP and downregulated hepatic TLR2 expression and its downstream signaling node MyD88. Consequently, GML ameliorated systemic inflammation in HFD-fed mice, which was related to the reduced circulating LPS load.

Various studies have reported that gut microbiota dysbiosis participates in obesity and related metabolic complications (43). The gut microbiota diversity has been linked to obesity, as obese subjects show a less diverse composition of their gut microbiotas (47). Concomitantly with the metabolic modulation by GML supplementation, a significantly changed composition and increased diversity of the gut microbiota were also observed in GML-treated HFD-fed mice. It has been reported that the increased ratio of *Firmicutes* to *Bacteroidetes* (F/B) in HFD-fed mice plays an important part in energy harvesting and the pathogenesis of obesity (48). However, alterations in the F/B ratio vary in different studies. The results of Bajzer and Seeley indicated that the gut microbiota exerts limited effects on energy extraction (49), and Zhang et al. showed that the F/B ratio was not correlated with body weight or adiposity percentage (50). In our study, we found an increased abundance of *Firmicutes* and a decreased abundance of *Bacteroidetes* (implying a higher F/B ratio) in GML-treated HFD-fed mice. Several factors affect the gut microbiota, and different experimental designs and conditions might lead to contradictory observations. Additionally, GML supplementation reversed HFD-induced higher proportions of *Proteobacteria* and lower proportions of *Verrucomicrobia*. Previous studies indicated that the phylum *Proteobacteria* is associated with LPS production and that the phylum *Verrucomicrobia* is related to LPS suppression (51). Such changes induced by GML treatment were consistent with its modulatory effects on the levels of circulating LPS and LBP. Furthermore, the same changes in *Proteobacteria* and *Verrucomicrobia* were also observed in our previous study on the effects of low-dose GML treatment in LFD-fed mice (20).

Our observations demonstrated that GML supplementation decreased the abundances of organisms in the *Bacteroides*, *Dorea*, *Eggerthella* (including *E. lenta*), and *Parabacteroides* genera. Consistently, a previous study showed that hepatic steatosis results in the enrichment of *Bacteroides* organisms, which is associated with insulin resistance (52). The research of Wang et al. showed that *Dorea* presented a positive correlation with metabolic syndrome phenotypes (53). In addition, higher levels of *Eggerthella* organisms (especially *E. lenta*) and *Parabacteroides* organisms were detected in individuals with type 2 diabetes than in healthy individuals (54, 55). Collectively, we speculated that the metabolic effects of GML were partially due to the decreased abundances of these microbes, which are positively related to obesity and metabolic disorders. Moreover, GML supplementation enhanced the abundances of *Bifidobacterium* and *B. pseudolongum* organisms. Previous studies reported that *Bifidobacterium* ameliorates obesity, inflammation, and associated metabolic disorders, and its lower abundance is associated with obesity and diabetes at various stages of life (7, 56). The loss of gut *Bifidobacterium* organisms has been regarded as a typical association with metabolic endotoxemia, which is related to mucosal barrier function and inhibition of pathogen bacterial adhesion (57, 58). The increased proportions of *Bifidobacterium* spp. induced by prebiotics resulted in improvements in gut barrier function, circulating LPS level, and hepatic inflammation and oxidation (59, 60). The results of Liu et al. indicated that HFD led to a 5-fold decrease in the abundance of *B. pseudolongum* (61), and similar results were also found in the studies of Schott et al. and Wang et al. (62, 63). Administration of *B. pseudolongum* decreased gamma interferon production and increased IL-10 production in lymph node cells (64). Consistently with these observations, the correlation analysis between gut bacteria and specific metabolic disorder parameters in our study indicated that the abundance of *B. pseudolongum* was negatively correlated with serum LPS and LBP levels, visceral fat deposition, systemic inflammation, and insulin resistance. The improvements in serum hyperlipidemia by GML supplementation were also correlated with an increased level of *B. pseudolongum* organisms. Furthermore, the significantly changed serum metabolites LPC(18:0), hexanoylglycine, and suberylglycine were also closely associated with *B. pseudolongum*, suggesting the potential influence of *B. pseudolongum* on host lipid metabolism. The negative correlation between *B. pseudolongum* and serum linolenic acid found in our study was supported by Gorissen et al., who demonstrated that *B. pseudolongum* was able to produce anti-obesity, conjugated α-linolenic acid isomers from free linoleic acid and α-linolenic acid (65). Importantly, the correlation analysis of the microbiome, metabolome, and transcriptome demonstrated that the abundance of *B. pseudolongum* was negatively correlated with glycerophospholipid metabolism, which implied that *B. pseudolongum* plays a key role in the GML-induced regulation of lipid metabolism in HFD-fed mice. Kindt et al. reported that the gut microbiota was closely related to host hepatic lipid metabolism, including glycerophospholipid species profiles (66).

Broad-spectrum antibiotics are used to evaluate the effects of the gut microbiota on the host (67). Our study first demonstrated that the prevention of obesity and related biochemical abnormalities by GML supplementation partially depended on modulating the gut microbiota. The significant differences between the gut microbiotas of the HFD control group and the GML-treated group were abolished after antibiotic treatment. Concomitantly, a depleted gut microbiota also counteracted almost all of the GML-induced metabolic improvements in an HFD-induced metabolic disorder.

### Conclusions.

Our results demonstrated that high-dose GML supplementation significantly modulated the gut microbiota composition of HFD-fed mice, resulting in anti-obesity and anti-inflammatory effects and improved glucose homeostasis, which were highly related to the increased abundance of Bifidobacterium pseudolongum. The improvements in lipid metabolism by GML treatment occurred mainly through reducing lipogenesis and promoting lipid β-oxidation and cholesterol metabolism, while glycerophospholipid metabolism was the most affected metabolic pathway. Collectively, our work indicates that GML has the potential to prevent obesity and related metabolic disorders.

## MATERIALS AND METHODS

### Animals and diets.

All experimental procedures were approved by the Committee on the Ethics of Animal Experiments of Zhejiang Chinese Medical University (approval no. ZSLL-2017-077). C57BL/6J male mice (6 weeks old, 15 per group) were purchased from Shanghai SLAC Laboratory Animal Co., Ltd. (China) and kept under controlled environmental conditions (12-h light/dark cycle, 23 to 26°C), with free access to food and water. After 2 weeks of acclimatization, mice were randomly divided into three groups and housed in groups of three animals per cage for 16 weeks. One group was fed a normal chow diet (NCD; 3,850 kcal/kg). The other two groups were fed an HFD (4,730 kcal/kg); one of these HFD-fed groups was regarded as a control group, and the other group was additionally treated with GML at 1,600 mg/kg of diet. To investigate the role of gut microbiota on host metabolism during GML (Tokyo Chemical Industry, Japan) intervention, C57BL/6 male mice (aged 6 weeks, 15 per group) were treated with antibiotics (metronidazole [1 g/liter; Sigma, USA], ampicillin [1 g/liter; Sigma, USA], neomycin [1 g/liter; Sigma, USA], and vancomycin [0.5 g/liter; Sigma, USA]) in drinking water for 10 days before being divided into 2 groups fed either an HFD (HFD-Abx) or an HFD supplemented with GML at 1,600 mg/kg of diet (HFD-GML-Abx) for 16 weeks. The details of the diets are listed in Table S2 in the supplemental material. Body weight and food intake were measured weekly. Fresh fecal samples were collected weekly, mice were placed in an empty autoclaved cage separately and allowed to defecate normally; the first two fecal pellets were collected and immediately stored at –80°C. Serum, liver, colon, epididymal, and subcutaneous inguinal adipose tissues were collected at week 16 and preserved at –80°C for further analysis. The detailed procedures of morphological analysis, RNA isolation, quantitative real-time PCR (qRT-PCR), and hepatic transcriptome analysis are provided in Text S1 in the supplemental material.

10.1128/mBio.00190-20.8 TABLE S2 Compositions of experimental diets. Download Table S2, DOCX file, 0.02 MB. Copyright © 2020 Zhao et al. 2020 Zhao et al. This content is distributed under the terms of the Creative Commons Attribution 4.0 International license.

10.1128/mBio.00190-20.9 TABLE S3 Spearman correlation coefficient between 6 GML-induced significantly changed serum metabolites and significantly changed gut microbes (at the family, genus, and species levels). Download Table S3, DOCX file, 0.02 MB. Copyright © 2020 Zhao et al. 2020 Zhao et al. This content is distributed under the terms of the Creative Commons Attribution 4.0 International license.

10.1128/mBio.00190-20.10 TEXT S1 Supplemental materials and methods. Download Text S1, DOCX file, 0.02 MB. Copyright © 2020 Zhao et al. 2020 Zhao et al. This content is distributed under the terms of the Creative Commons Attribution 4.0 International license.

### Biochemical analyses.

Serum glucose, TG, total cholesterol (TC), high-density lipoprotein cholesterol (HDLC), and low-density lipoprotein cholesterol (LDLC) levels were determined with commercial kits (Nanjing Jiancheng, China) based on the manufacturer’s instructions. Serum lipopolysaccharide (LPS), LPS-binding protein (LBP), and free fatty acids (FFA) (BioVision, USA), tumor necrosis factor alpha (TNF-α) and interleukin 6 (IL-6) (eBioscience, USA), and leptin and adiponectin (R&D Systems, USA) were measured using a commercial enzyme-linked immunosorbent assay (ELISA) kit.

### Gut microbiota analysis.

Bacterial genomic DNA was extracted from frozen collected fecal samples stored at −80°C using a QIAamp DNA stool minikit (Qiagen, Germany) according to the manufacturer’s instructions. Next-generation sequencing was performed on an Illumina HiSeq system. The detailed procedure of library construction and sequencing is presented in Text S1. To compare α-diversities, the operational taxonomic unit (OTU) was rarified to several metrics, and the observed species, Chao 1 index, Simpson index, and Shannon index were calculated. For β-diversity analysis, the weighted UniFrac distance and unweighted UniFrac distance were calculated and used in a principal-coordinate analysis (PCoA). Differences in gut microbiota compositions among groups were identified by QIIME software, v1.8.0. R 3.1.0 was used for analyzing and graphing the gut microbiota profiles.

### Untargeted UHPLC-Q-TOF-MS metabolite profiling analysis.

The details of serum metabolite measurement and analysis are provided in Text S1. SIMCA-P 14.1 (Umetrics, Sweden) was used for multivariate statistical calculations and plotting of serum metabolite profiles. The distribution of origin data and general separation were performed and demonstrated by an unsupervised principal-component analysis (PCA). The supervised orthogonal projection to latent structure discriminant analysis (OPLS-DA) models were validated by 7-fold cross-validation and 200 permutation tests. The metabolic features with a variable importance in projection (VIP) value of >1.0 and a *P* (corrected) of >0.5 in the OPLS-DA model were considered to be significantly different metabolites in the paired comparison.

### Statistical analysis.

Data are expressed as means ± standard errors of the means (SEM). Statistical differences between more than two groups were assessed by one-way analysis of variance (ANOVA) or two-way ANOVA, followed by Tukey’s multiple-comparison posttests or by the nonparametric factorial Kruskal-Wallis test with unpaired Wilcoxon comparison test. Differences between two groups were determined by an unpaired two-tailed Student *t* test. Statistical analysis was performed using GraphPad Prism 6.0 (GraphPad Software, Inc., USA), and a *P* of <0.05 was noted as significant. Correlations between variables were identified by Spearman’s correlation.

### Availability of data.

All the relevant data which support our findings are available upon reasonable request; some have already been included here and in the supplemental material. Microbiota sequencing data which support the findings in our study have been deposited into NCBI’s Sequence Read Archive under accession number PRJNA506598. mRNA sequencing data that support our findings have been deposited in NCBI’s Sequence Read Archive under accession number PRJNA507884.
