# Supplementary material for: Modulation of the Gut Microbiota during High-Dose Glycerol Monolaurate-Mediated Amelioration of Obesity in Mice Fed a High-Fat Diet
Source: mBio. 2020 Apr 7;11(2):e00190-20. doi: 10.1128/mBio.00190-20 (PMC7157765; doi:10.1128/mBio.00190-20)
Supplement: FIG S1 [file mBio.00190-20-sf001.docx]

**Supplementary Figure S1**


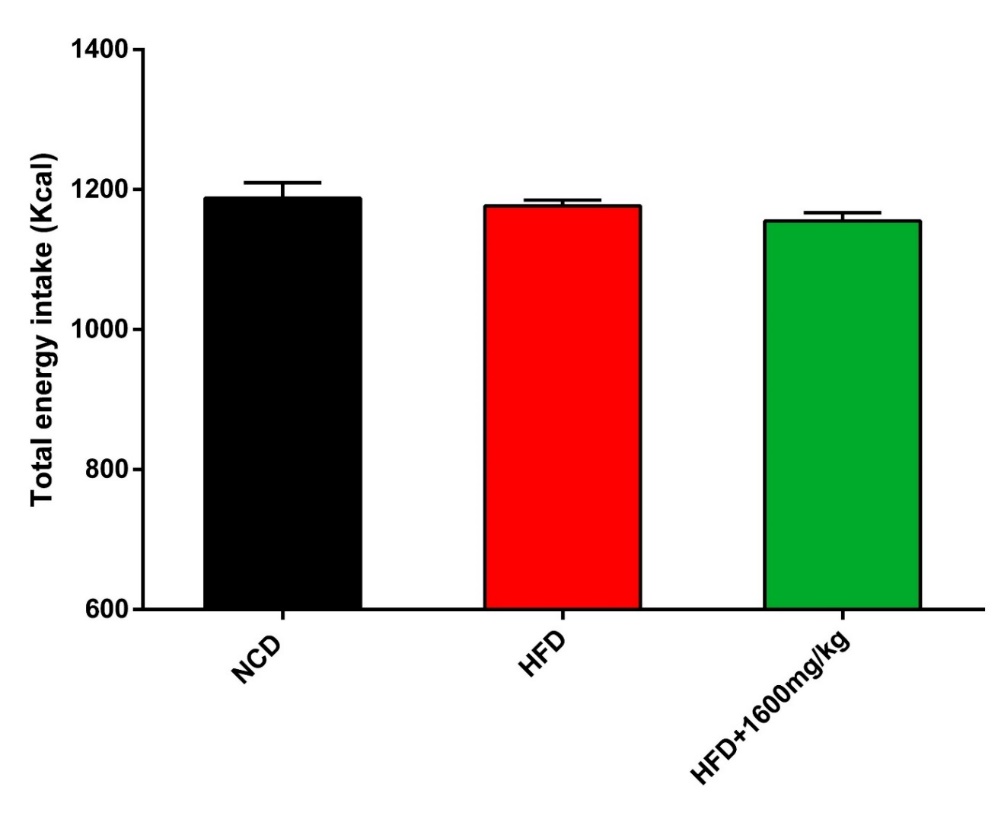


**Supplementary Figure S1 Total energy intake is not significantly altered by GML supplementation** (n = 15 for each group)**.**
