# Supplementary material for: Modulation of the Gut Microbiota during High-Dose Glycerol Monolaurate-Mediated Amelioration of Obesity in Mice Fed a High-Fat Diet
Source: mBio. 2020 Apr 7;11(2):e00190-20. doi: 10.1128/mBio.00190-20 (PMC7157765; doi:10.1128/mBio.00190-20)
Supplement: TABLE S1 [file mBio.00190-20-st001.docx]

### Supplementary Table S1. Lipid profile of feces of the experimental groups using the Folch method ([1](#_ENREF_1)).

|  | **NCD** | **HFD** | **HFD+1600 mg/kg GML** |
| --- | --- | --- | --- |
| Fecal total lipids% | 6.85±1.37 | 14.95±1.46^##^ | 15.45±1.48^##^ |

NCD, mice fed a normal chow diet; HFD, mice fed a high fat diet; HFD+1600 mg/kg, mice fed a high fat diet with 1600 mg/kg GML; Values with asterisks and pound symbols are significantly different based on one-way analysis of variance (ANOVA) with Tukey’s post hoc test (**p* < 0.05 versus HFD controls, ***p* < 0.01 versus HFD controls, ****p* < 0.001 versus HFD controls; #*p* < 0.05 versus NCD controls, ##*p* < 0.01 versus NCD controls, ###*p* < 0.001 versus NCD controls), n = 8.

1. Folch J, Lees M, Stanley GHS. 1957. A Simple Method for the Isolation and Purification of Total Lipides from Animal Tissues. J Biol Chem 226:497-509.
