# Supplementary material for: Modulation of the Gut Microbiota during High-Dose Glycerol Monolaurate-Mediated Amelioration of Obesity in Mice Fed a High-Fat Diet
Source: mBio. 2020 Apr 7;11(2):e00190-20. doi: 10.1128/mBio.00190-20 (PMC7157765; doi:10.1128/mBio.00190-20)
Supplement: FIG S2 [file mBio.00190-20-sf002.docx]

**
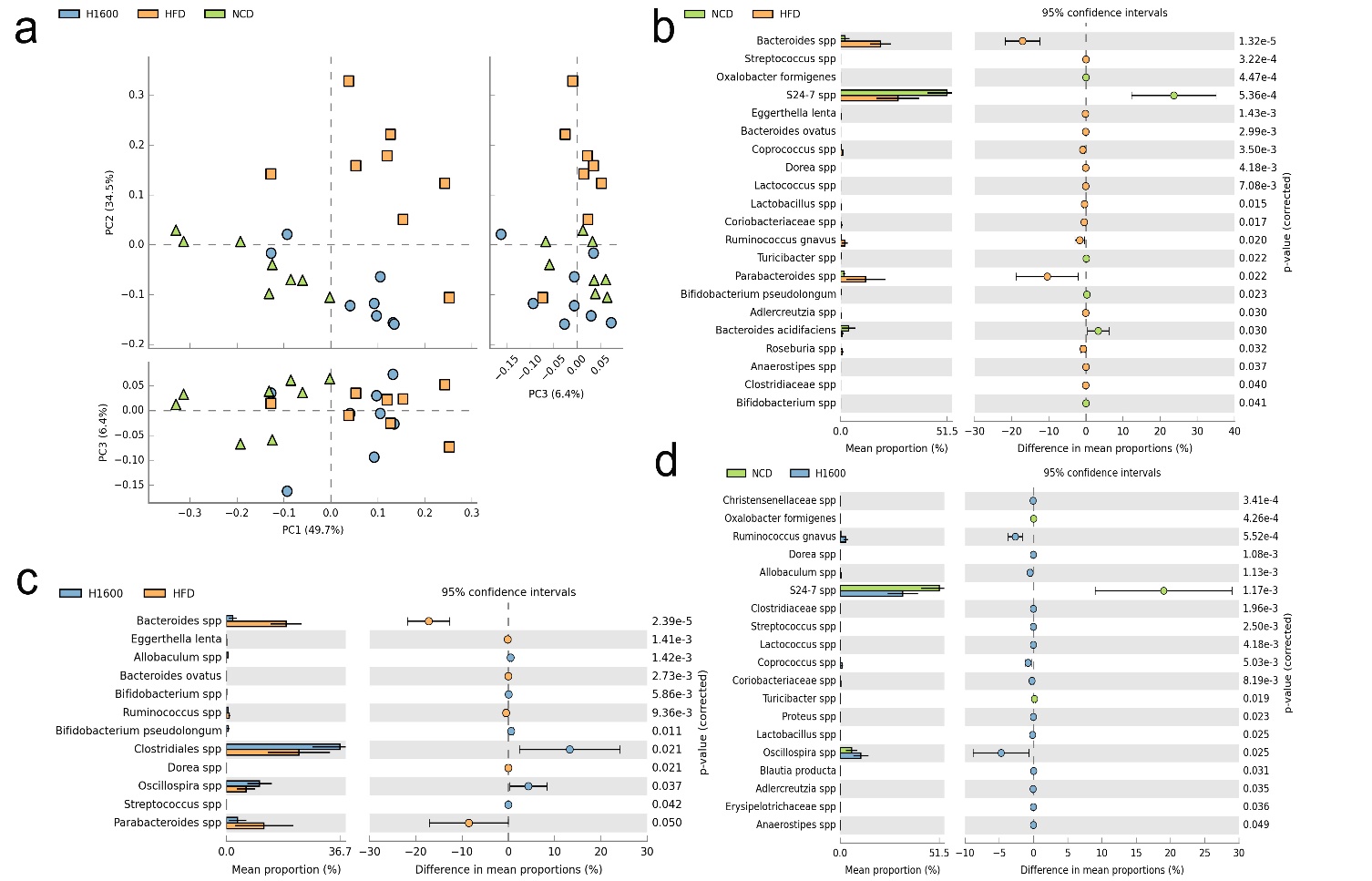
Supplementary Figure S2**

**Supplementary Figure S2 STAMP analysis of gut microbiota composition at species level among NCD, HFD and HFD + 1600 ppm GML (H1600) groups.**

**a** PCA plot comparing genus-level taxonomic profiles (n = 10 for each group). Plots showing significant differences in abundance of species between **b** NCD and HFD groups, **c** HFD and H1600 groups and **d** NCD and H1600 groups, the dot plots on the right side display the difference in mean proportions between the two groups compared with associated p-value, the bar graphs on the left side demonstrated the mean proportion of genus in each group.
