# Supplementary material for: Modulation of the Gut Microbiota during High-Dose Glycerol Monolaurate-Mediated Amelioration of Obesity in Mice Fed a High-Fat Diet
Source: mBio. 2020 Apr 7;11(2):e00190-20. doi: 10.1128/mBio.00190-20 (PMC7157765; doi:10.1128/mBio.00190-20)
Supplement: FIG S3 [file mBio.00190-20-sf003.docx]

**Supplementary Figure S3**


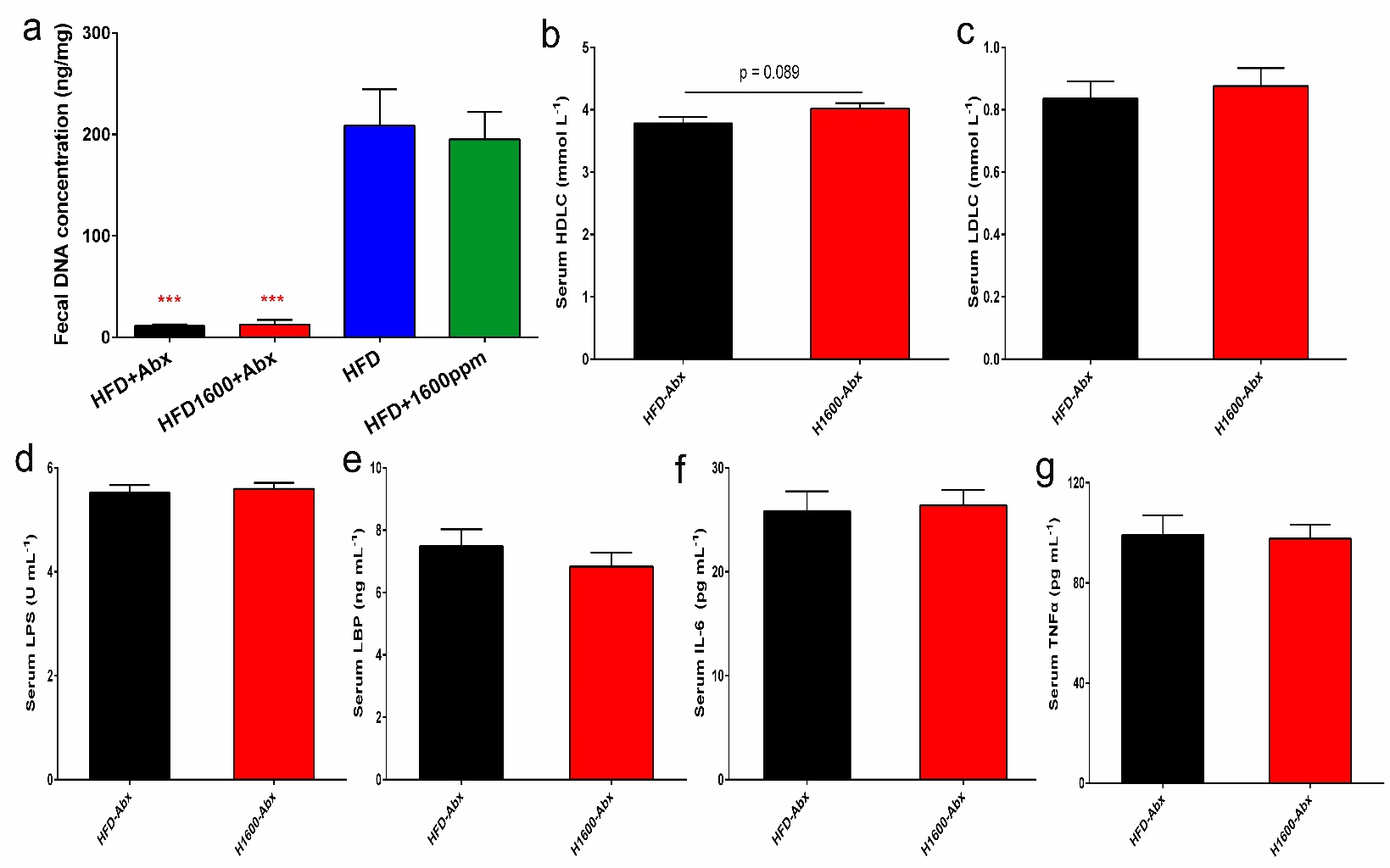


**Supplementary Figure S3 Continuous antibiotic treatment abrogated the metabolic improvement induced by GML supplementation in HFD-fed mice. Mice were fed a HFD supplemented with (H1600-Abx) or without 1600 mg/kg GML (HFD-Abx) under antibiotic treatment for 16 weeks. a** Continuous antibiotics treatment significantly reduced the abundance of gut microbes, supported by the DNA concentrations in fecal samples (n = 10 for each group). The GML-treated group exhibited no significant difference in **b** serum HDLC, **c** serum LDLC, **d** serum LPS, **e** serum LBP, **f** serum IL-6 and **g** serum TNF-α, n =14-15 (**b**-**g**). Data are expressed as the mean ± SEM. Values with asterisks are significantly different based on one-way analysis of variance with Tukey’s post hoc test (*p < 0.05 versus HFD controls, **p < 0.01 versus HFD controls, ***p < 0.001 versus HFD controls).
