# Supplementary material for: Modulation of the Gut Microbiota during High-Dose Glycerol Monolaurate-Mediated Amelioration of Obesity in Mice Fed a High-Fat Diet
Source: mBio. 2020 Apr 7;11(2):e00190-20. doi: 10.1128/mBio.00190-20 (PMC7157765; doi:10.1128/mBio.00190-20)
Supplement: FIG S4 [file mBio.00190-20-sf004.docx]

**Supplementary Figure S4**


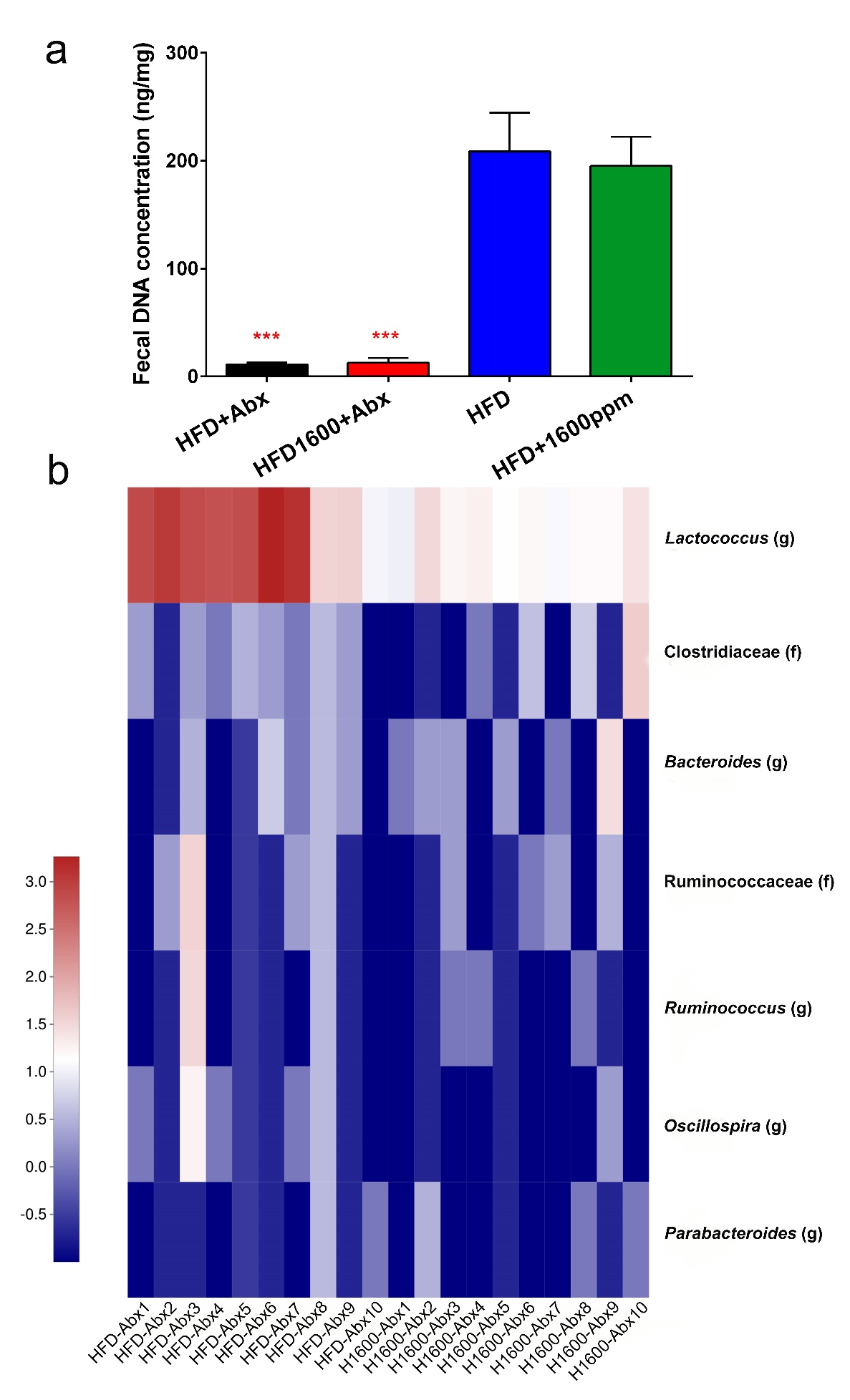


**Supplementary Figure S4 Continuous antibiotics treatment abolished the gut microbiota alterations induced by GML supplementation. a** Continuous antibiotics treatment significantly reduced the abundance of gut microbes, supported by the DNA concentrations in fecal samples (n = 10 for each group). **b** Heatmap demonstrated the abundance of detected bacterial taxa which were significantly changed by GML treatment in HFD-fed mice after antibiotics treatment. Data are expressed as the mean ± SEM. Values with asterisk are significantly different based on one-way analysis of variance with Tukey post hoc test (*p < 0.05 verus HFD controls, **p < 0.01 verus HFD controls, ***p < 0.001 verus HFD controls)
