# Supplementary material for: Modulation of the Gut Microbiota during High-Dose Glycerol Monolaurate-Mediated Amelioration of Obesity in Mice Fed a High-Fat Diet
Source: mBio. 2020 Apr 7;11(2):e00190-20. doi: 10.1128/mBio.00190-20 (PMC7157765; doi:10.1128/mBio.00190-20)
Supplement: FIG S5 [file mBio.00190-20-sf005.docx]

**Supplementary Figure S5**

**
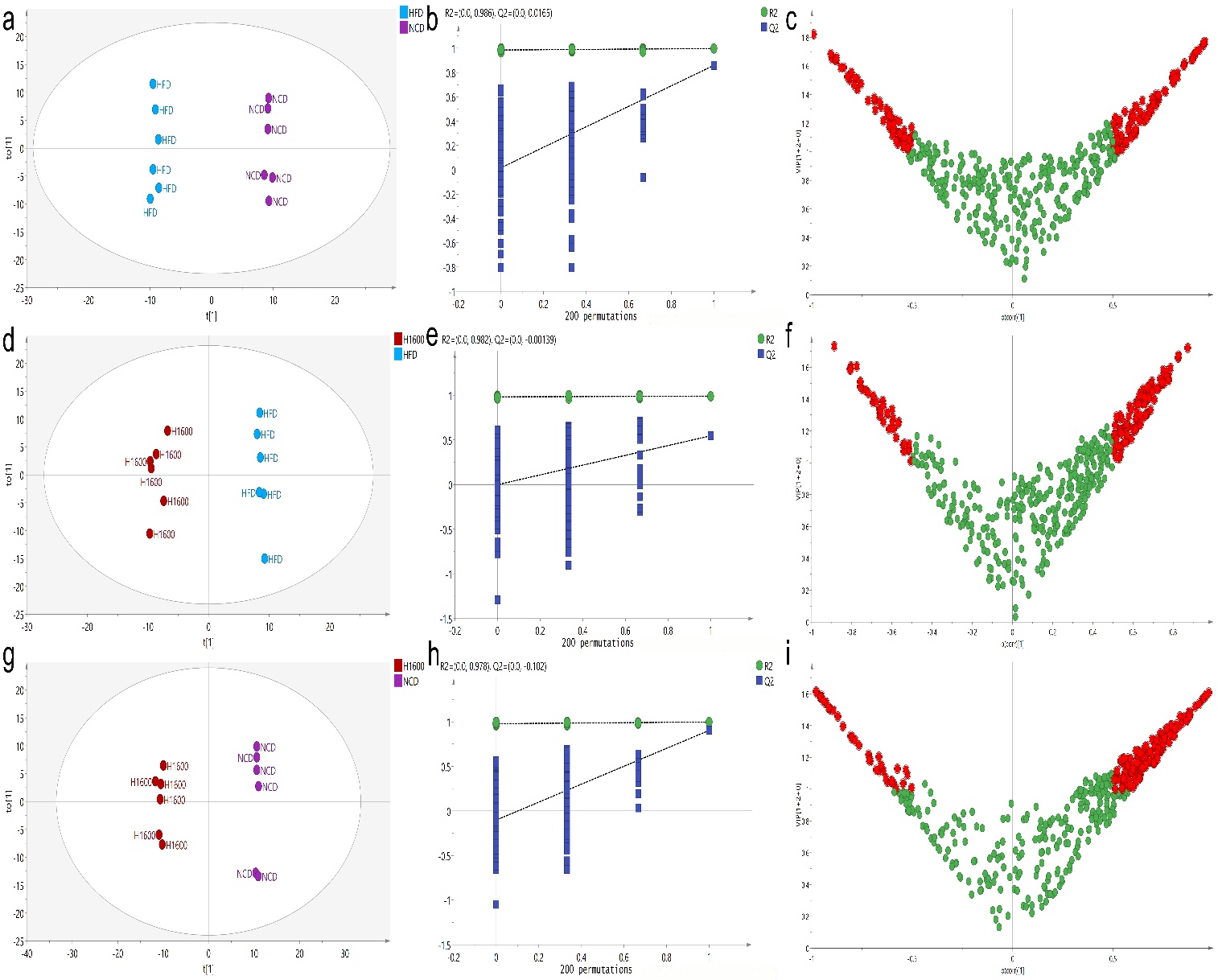
**

**Supplementary Figure S5 Preliminary identification of potential biomarkers of serum metabolomics in ESI+ mode.** OPLS-DA model built to discriminate between NCD and HFD groups in ESI+ mode **a** Scores plot, **b** Permutation tests for the goodness-of-fit (R^2^) and goodness-of-prediction (Q^2^) parameters obtained from a sevenfold cross-validated O-PLS regression model, **c** VIP loading plot (The red spots represented significantly differential metabolites with VIP > 1.0 and p (corr) > 0.5). OPLS-DA model built to discriminate between HFD and H1600 groups in ESI+ mode **d** Scores plot, **e** Permutation tests for the goodness-of-fit (R^2^) and goodness-of-prediction (Q^2^) parameters obtained from a sevenfold cross-validated O-PLS regression model, **f** VIP loading plot (The red spots represented significantly differential metabolites with VIP > 1.0 and p (corr) > 0.5). OPLS-DA model built to discriminate between NCD and H1600 groups in ESI+ mode **g** Scores plot, **h** Permutation tests for the goodness-of-fit (R^2^) and goodness-of-prediction (Q^2^) parameters obtained from a sevenfold cross-validated O-PLS regression model, **i** VIP loading plot (The red spots represented significantly differential metabolites with VIP > 1.0 and p (corr) > 0.5).
