# Supplementary material for: Modulation of the Gut Microbiota during High-Dose Glycerol Monolaurate-Mediated Amelioration of Obesity in Mice Fed a High-Fat Diet
Source: mBio. 2020 Apr 7;11(2):e00190-20. doi: 10.1128/mBio.00190-20 (PMC7157765; doi:10.1128/mBio.00190-20)
Supplement: TABLE S2 [file mBio.00190-20-st002.docx]

### Supplementary Table S2. Compositions of experimental diets

| **Ingredients (g / 100 g diet)** | **Normal chow diet** | **High fat diet** |
| --- | --- | --- |
| Casein | 18.96 | 23.31 |
| L-Cystine | 0.28 | 0.35 |
| Corn Starch | 29.86 | 8.48 |
| Maltodextrin | 3.32 | 11.65 |
| Sucrose | 33.17 | 20.14 |
| Cellulose | 4.74 | 5.83 |
| Soybean Oil | 2.37 | 2.91 |
| Lard | 1.90 | 20.68 |
| Mineral Mix | 2.68 | 3.31 |
| Potassium Citrate, 1 H_2_O | 1.56 | 1.92 |
| Vitamin Mix | 0.95 | 1.16 |
| Choline Bitartrate | 0.19 | 0.23 |
| **Calories supplementation (kcal %)** |  |  |
| Proteins | 20 | 20 |
| Carbohydrates | 70 | 35 |
| Fats | 10 | 45 |
| **Total calories (kcal / 100 g diet)** | 385 | 473 |
