# Supplementary material for: Modulation of the Gut Microbiota during High-Dose Glycerol Monolaurate-Mediated Amelioration of Obesity in Mice Fed a High-Fat Diet
Source: mBio. 2020 Apr 7;11(2):e00190-20. doi: 10.1128/mBio.00190-20 (PMC7157765; doi:10.1128/mBio.00190-20)
Supplement: TABLE S3 [file mBio.00190-20-st003.docx]

### Supplementary Table S3. Spearman correlation coefficient between GML induced significantly changed 6 serum metabolites and significantly changed gut microbes (at family, genus and species levels)

| **Gut microbe** | **Serum metabolite** | **Spearman correlation coefficient** | ***p* value** |
| --- | --- | --- | --- |
| *Ruminococcus* | PC(18:1/18:1) | 0.64 | 0.021 |
| *Allobaculum* | PC(18:1/18:1) | 0.63 | 0.005 |
| *Streptococcus* | PC(18:1/18:1) | 0.53 | 0.020 |
| *Ruminococcus* | Hexanoylglycine | -0.50 | 0.035 |
| *Bacteroides* | Hexanoylglycine | -0.53 | 0.025 |
| *Bifidobacterium* | Hexanoylglycine | 0.54 | 0.020 |
| *Dorea* | Hexanoylglycine | -0.73 | 0.001 |
| *Eggerthella* | Hexanoylglycine | -0.64 | 0.004 |
| *Parabacteroides* | Hexanoylglycine | -0.63 | 0.006 |
| *Streptococcus* | Hexanoylglycine | -0.51 | 0.031 |
| *Eggerthella lenta* | Hexanoylglycine | -0.64 | 0.004 |
| *Bacteroides ovatus* | Hexanoylglycine | -0.64 | 0.004 |
| *Bifidobacterium pseudolongum* | Hexanoylglycine | 0.54 | 0.020 |
| Clostridiaceae | Hexanoylglycine | -0.81 | 0.000 |
| Mogibacteriaceae | Hexanoylglycine | -0.82 | 0.000 |
| *Ruminococcus* | Suberylglycine | -0.56 | 0.017 |
| *Bacteroides* | Suberylglycine | -0.64 | 0.005 |
| *Bifidobacterium* | Suberylglycine | 0.67 | 0.002 |
| *Dorea* | Suberylglycine | -0.76 | 0.000 |
| *Eggerthella* | Suberylglycine | -0.75 | 0.000 |
| *Parabacteroides* | Suberylglycine | -0.53 | 0.025 |
| *Streptococcus* | Suberylglycine | -0.57 | 0.014 |
| *Eggerthella lenta* | Suberylglycine | -0.75 | 0.000 |
| *Bacteroides ovatus* | Suberylglycine | -0.73 | 0.001 |
| *Bifidobacterium pseudolongum* | Suberylglycine | 0.67 | 0.002 |
| Clostridiaceae | Suberylglycine | -0.75 | < 0.001 |
| Mogibacteriaceae | Suberylglycine | -0.86 | < 0.001 |
| *Ruminococcus* | Stearoylcarnitine | 0.63 | 0.007 |
| *Allobaculum* | Stearoylcarnitine | 0.70 | 0.002 |
| *Streptococcus* | Stearoylcarnitine | 0.61 | 0.007 |
| *Ruminococcus* | Lathosterol | 0.72 | 0.001 |
| *Allobaculum* | Lathosterol | 0.73 | 0.001 |
| *Streptococcus* | Lathosterol | 0.62 | 0.006 |
| *Ruminococcus* | LysoPC(18:0) | 0.57 | 0.015 |
| *Bacteroides* | LysoPC(18:0) | 0.63 | 0.007 |
| *Bifidobacterium* | LysoPC(18:0) | -0.58 | 0.011 |
| *Dorea* | LysoPC(18:0) | 0.69 | 0.001 |
| *Eggerthella* | LysoPC(18:0) | 0.68 | 0.002 |
| *Parabacteroides* | LysoPC(18:0) | 0.57 | 0.015 |
| *Streptococcus* | LysoPC(18:0) | 0.53 | 0.023 |
| *Eggerthella lenta* | LysoPC(18:0) | 0.68 | 0.002 |
| *Bacteroides ovatus* | LysoPC(18:0) | 0.74 | < 0.001 |
| *Bifidobacterium pseudolongum* | LysoPC(18:0) | -0.58 | 0.011 |
| Clostridiaceae | LysoPC(18:0) | 0.67 | 0.002 |
| Mogibacteriaceae | LysoPC(18:0) | 0.77 | < 0.001 |
