# Supplementary material for: Modulation of the Gut Microbiota during High-Dose Glycerol Monolaurate-Mediated Amelioration of Obesity in Mice Fed a High-Fat Diet
Source: mBio. 2020 Apr 7;11(2):e00190-20. doi: 10.1128/mBio.00190-20 (PMC7157765; doi:10.1128/mBio.00190-20)
Supplement: TEXT S1 [file mBio.00190-20-s0001.docx]

# SUPPLEMENTARY MATERIALS

## Supplementary methods

### Glucose homeostasis analysis

An intraperitoneal glucose tolerance test (IGTT) was performed during the fifteenth week of the experiment, mice were fasted overnight and administered glucose by intraperitoneal injection (2 g/kg body weight), and blood glucose levels were measured by a glucose meter (Roche Diagnostics, Switzerland) using whole blood collected from the tip of the tail vein.

The serum samples collected at week 16 were used for fast glucose test (determined by a commercial kit, Nanjing Jiancheng, China) and fasting insulin test (determined by a commercial ELISA kit, Clound-Clone Corp, USA). The homeostasis model assessment of insulin resistance (HOMA-IR) was evaluated based on the following formula:

HOMA-IR = serum glucose (mmol/L) × serum insulin (μmol/mL) / 22.5

### Morphological analysis

Freshly isolated liver and epididymal adipose tissues were fixed in 10% formalin (neutral buffered), followed by embedding in paraffin, sectioning at 5 μm and staining with hematoxylin and eosin (H&E). Frozen liver sections (6 μm) were stained with oil red to display the hepatic lipid accumulation. Transmission electron microscopy (TEM) was used to further analyze the size of lipid drops in hepatic cells. Liver tissues were cut into 1 mm3 fragments and fixed with 2.5% glutaraldehyde in phosphate buffer (0.1 M, pH 7.0) overnight at 4°C. After that, it was washed in phosphate buffer for three times followed by post fixed with 1% OsO4 in 0.1 M phosphate buffer (pH 7.0) for 2 h and then washed three times by phosphate buffer. Tissues were dehydrated through graded alcohols (50, 70, 80, 90, 95 and 100%) for 20 min at each step and then transeferred to absolute acetone for 20 min. The specimens were infiltratedby acetone and Spurr resin for overnight and embedded in Spurr resin at last. LEICA EM UC7 were used to cut the specimens at 1.5 μm, sections were stained by uranyl acetate and alkaline lead citrate for 5 and 10 min respectively and examined with a Hitachi Model H-7650 TEM (Hitachi, Japan). The lipid area in oil red staining and the cell size of adipose tissues were quantified using Image-Pro Plus 6.1 (Media Cybernetics, Inc., USA).

### RNA isolate, quantitative real-time PCR (qRT-PCR) and hepatic transcriptome analysis

Liver total RNA was extracted using TRIzol reagent (Invitrogen, USA) according to the manufacturer’s instructions. cDNA was prepared by reverse transcription using a HiScript® Reverse Transcription Kit (Vazyme, China). SYBR Green qRT-PCR was performed on Roche LightCycler® 480 (LC480) system (Roche Applied Science, USA) according to the manufacturer's instructions (Vazyme, Nanjing, China). Values were normalized to RPS18 and calculated based on the 2^-△△Ct^ method. The detailed primer sequences used in qRT-PCR are listed in Supplementary Table S3. For hepatic transcriptome analysis, Total RNA of each liver sample was extracted using TRIzol Reagent (Invitrogen), quantified and qualified by Agilent 2100 Bioanalyzer (Agilent Technologies, Palo Alto, CA, USA), NanoDrop (Thermo Fisher Scientific Inc., Wilminton, USA) and 1% agrose gel. Next generation sequencing library preparations were constructed according to the manufacturer’s protocol (NEBNext® Ultra™ RNA Library Prep Kit for Illumina®). Then libraries with different indices were multiplexed and loaded on an Illumina HiSeq instrument according to manufacturer’s instructions (Illumina, San Diego, CA, USA). Sequencing was carried out using a 2 × 150bp paired-end (PE) configuration; image analysis and base calling were conducted by the HiSeq Control Software (HCS) + OLB + GAPipeline-1.6 (Illumina) on the HiSeq instrument. To obtain high quality clean data, filtering was procecssed by Trimmomatic (v0.30) and then mappped with reference genome sequences via software Hisat2 (v2.0.1). Differential expression analysis used the DESeq Bioconductor package, P value of genes and FDR (q value) were setted <0.05 to detect differential expressed ones. GO-TermFinder was used identifying Gene Ontology (GO) terms that annotate a list of enriched genes with a significant p-value less than 0.05. Enrich significant differential expression genes in KEGG (Kyoto Encyclopedia of Genes and Genomes) pathways were also annotated. The sequences were processed and analyzed by GENEWIZ (Suzhou, China). Gene co-expression modules were identified using the weighted gene co-expression network analysis (WGCNA) package for R.

**Gut microbiota analysis**

The quality of the libraries was confirmed using a NanoDrop 2000 spectrophotometer (Thermo, USA) and a 2% agarose gel. The V3-V4 region of the 16S rRNA gene from genomic DNA samples was amplified by PCR using the primers 341F and 806R (5’-ACTCCTACGGGRSGCAGCAG-3’, 5’-GGACTACVVGGGTATCTAATC-3’). The raw paired-end reads assembled by PANDAseq were quality-filtered, and then, the chimera and singletons were discarded by USEARCH. After these processes, high-quality reads were obtained for bioinformatics analysis and clustered into operational taxonomic units (OTUs) at ≥ 97% similarity according to USEARCH. A representative sequence of each OTU was selected, and taxonomic information was annotated using the Greengenes reference database.

### Untargeted UHPLC-QTOF-MS analysis and data processing

Collected serum samples were thawed at 4 ℃ on ice. Fifty microliters of sample was extracted with 150 μL of methanol, and 10 μL of internal standard (L-2-chlorophenylalanine) substance was added, followed by vortexing for 30 s，treating with ultrasound for 10 min (incubated in ice water), and incubating for 1 h at -20 ℃ to precipitate proteins. Then, the samples were centrifuged at 13,000 rpm for 15 min at 4 ℃. The supernatants were collected for UHPLC-QTOF-MS analysis. In addition, 20 μL of each sample supernatant was pooled as quality-control (QC) samples. At the beginning of each run, a batch of 5 injections of a QC sample was used to condition the column.

The UHPLC-QTOF-MS system (Agilent Technologies, Germany ) was consisted of a 1290 UHPLC system equipped with a ACQUITY UPLC^®^BEH C18 column (1.7 µm × 2.1 mm × 100 mm, Waters, USA) coupled to a TripleTOF 6600 mass spectrometer (AB Sciex, USA). The injection volume was 1μL. The Triple TOF mass spectrometer was used to acquire MS/MS spectra on an information-dependent basis (IDA), the acquisition software (Analyst TF 1.7, AB Sciex) continuously evaluated the full scan survey MS data as it collected and triggered the acquisition of MS/MS spectra depending on preselected criteria. In each cycle, precursor ions whose intensity greater than 100 were filtered for fragmentation at collision energy (CE) of 30 V (15 MS/MS events with product ion accumulation time of 50 msec each). Electrospray ionization (ESI) source conditions were set as following: Ion source gas 1 as 60 Psi, Ion source gas 2 as 60 Psi, Curtain gas as 35 Psi, source temperature 650℃, Ion Spray Voltage Floating (ISVF) was set to 5000 V for positive and 4000 V for negative ionization mode.

Raw MS raw datas were converted to the mzXML format using ProteoWizard, and processed by R package XCMS (version 3.2). The preprocessing results generated a data matrix that consisted of the retention time (RT), massto-charge ratio (m/z) values, and peak intensity. R package CAMERA was used for peak annotation after XCMS data processing. Authoritative databases were applied in metabolites identification, Human Metabolome Database: <http://www.hmdb.ca>, METLIN: <http://metlin.scripps.edu>, and Lipid MAPS: <http://www.lipidmaps.org>.
